# *Clostridium butyricum* and carbohydrate active enzymes contribute to the reduced fat deposition in pigs

**DOI:** 10.1002/imt2.160

**Published:** 2024-01-03

**Authors:** Lingyan Ma, Shiyu Tao, Tongxing Song, Wentao Lyu, Ying Li, Wen Wang, Qicheng Shen, Yan Ni, Jiang Zhu, Jiangchao Zhao, Hua Yang, Yingping Xiao

**Affiliations:** ^1^ State Key Laboratory for Managing Biotic and Chemical Threats to the Quality and Safety of Agro‐Products, Institute of Agro‐product Safety and Nutrition Zhejiang Academy of Agricultural Sciences Hangzhou China; ^2^ Department of Animal Nutrition and Feed Science, College of Animal Sciences and Technology Huazhong Agricultural University Wuhan China; ^3^ Guangdong Provincial Key Laboratory of Animal Molecular Design and Precise Breeding, College of Life Science and Engineering Foshan University Foshan China; ^4^ The Children's Hospital, Zhejiang University School of Medicine National Clinical Research Center for Child Health Hangzhou China; ^5^ Department of Animal Science, Division of Agriculture University of Arkansas Fayetteville Arkansas USA

**Keywords:** CAZymes, *Clostridium butyricum*, fatness, gut microbiota, Jinhua pigs, metagenomics

## Abstract

Pig gastrointestinal tracts harbor a heterogeneous and dynamic ecosystem populated with trillions of microbes, enhancing the ability of the host to harvest energy from dietary carbohydrates and contributing to host adipogenesis and fatness. However, the microbial community structure and related mechanisms responsible for the differences between the fatty phenotypes and the lean phenotypes of the pigs remained to be comprehensively elucidated. Herein, we first found significant differences in microbial composition and potential functional capacity among different gut locations in Jinhua pigs with distinct fatness phenotypes. Second, we identified that Jinhua pigs with lower fatness exhibited higher levels of short‐chain fatty acids in the colon, highlighting their enhanced carbohydrate fermentation capacity. Third, we explored the differences in expressed carbohydrate‐active enzyme (CAZyme) in pigs, indicating their involvement in modulating fat storage. Notably, *Clostridium butyricum* might be a representative bacterial species from Jinhua pigs with lower fatness, and a significantly higher percentage of its genome was dedicated to CAZyme glycoside hydrolase family 13 (GH13). Finally, a subsequent mouse intervention study substantiated the beneficial effects of *C. butyricum* isolated from experimental pigs, suggesting that it may possess characteristics that promote the utilization of carbohydrates and hinder fat accumulation. Remarkably, when Jinhua pigs were administered *C. butyricum*, similar alterations in the gut microbiome and host fatness traits were observed, further supporting the potential role of *C. butyricum* in modulating fatness. Taken together, our findings reveal previously overlooked links between *C. butyricum* and CAZyme function, providing insight into the basic mechanisms that connect gut microbiome functions to host fatness.

## Highlight

- Significant differences in microbial composition and potential functional capacity among different gut locations were found in Jinhua pigs with distinct fatness phenotypes.
- Jinhua pigs with lower fatness exhibited higher levels of short‐chain fatty acids in the colon, highlighting their enhanced carbohydrate fermentation capacity.
- *Clostridium butyricum* might be a representative bacterial species from Jinhua pigs with lower fatness, and a significantly higher percentage of its genome was dedicated to CAZyme glycoside hydrolase family 13 (GH13).
- Isolated *C. butyricum* alleviated fat accumulation and increased the enrichment of GH13 in obese mice.

## Graphical abstract

In the present study, we found significant differences in microbial composition and potential functional capacity among different gut locations in Jinhua pigs with distinct fatness phenotypes. Second, we identified that Jinhua pigs with lower fatness exhibited higher levels of short‐chain fatty acids in the colon, highlighting their enhanced carbohydrate fermentation capacity. Notably, *Clostridium butyricum* might be a representative bacterial species from Jinhua pigs with lower fatness, and a significantly higher percentage of its genome was dedicated to CAZyme glycoside hydrolase family 13 (GH13). Finally, a subsequent mouse intervention study substantiated the beneficial effects of *C. butyricum* isolated from experimental pigs, suggesting that it may possess characteristics that promote the utilization of carbohydrates and hinder fat accumulation. Remarkably, when Jinhua pigs were administered *C. butyricum*, similar alterations in the gut microbiome and host fatness traits were observed, further supporting the potential role of *C. butyricum* in modulating fatness.

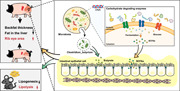

## INTRODUCTION

Over the past decade, extensive research has been dedicated to investigating the human gut microbiome and its intricate involvement in various diseases, thereby unveiling its profound impacts on metabolism, nutrition, physiology, and immune function [1, 2, 3, 4]. Pigs exhibit anatomical and physiological similarities to humans that surpass those observed in small rodents. This likeness positions pigs as an appealing and robust choice for modeling human diseases, consequently garnering increased attention toward porcine gut microbiome research [5, 6, 7]. Within the gastrointestinal (GI) tract of pigs thrives a diverse and dynamically intricate ecosystem hosting trillions of commensal microbes that interact with the host, thereby assuming a pivotal role in the maintenance of metabolic homeostasis [8, 9]. The continued advancement of pig microbiota studies is poised to serve as a vital wellspring of support, facilitating the progress of insights into human diseases over time.

Excessive lipid accumulation poses a substantial health risk to both the human and animal populations [10]. Furthermore, fatness has been regarded as a typically complex and economic trait in pig production owing to its implications for fattening efficiency, meat quality, reproductive performance, and immunity [11, 12]. Studies have suggested that the gut microbiota plays a role in fat accumulation and is correlated with the formation of adiposity [13, 14]. Moreover, our previous studies have confirmed a strong connection between the gut microbiome, lipid storage, and swine health across different breeds [15, 16, 17]. Given the critical role that the gut microbiota plays in swine health and production, it is crucial to better understand the function of the microbial communities in pigs and to identify beneficial bacterial strains involved in lipid deposition.

Local pig breeds exhibit a heightened degree of adaptation to their specific environmental conditions and food sources and, therefore, present a valuable resource. The Jinhua pig is mainly located in the Jinhua area of Zhejiang Province, China, and is a famous Chinese indigenous black‐and‐white swine species that is a traditional, slow‐growing breed with a high body fat content [18]. As a typical fatty pig breed, the Jinhua pig has emerged as an exceptional model for investigating the mechanisms of fat deposition. However, the microbial community structure and related mechanisms responsible for the differences between the fatty phenotypes and the lean phenotypes of the Jinhua pig breed remain to be comprehensively elucidated. The objective of the present article was to investigate the microbial community structure and potential functional capacity of the microbiome in Jinhua pigs with distinct fatness phenotypes, contributing to a better understanding of the mechanisms responsible for differences in fat deposition between pigs of different metabolic types. Our investigation pinpointed *Clostridium butyricum* as a prominent bacterial species within Jinhua pigs exhibiting lower levels of fatness and uncovered potential links between *C*. *butyricum* and carbohydrate‐active enzyme (CAZyme) function, further providing insight into the basic mechanisms that connect gut microbiota functions to host fatness.

## RESULTS

### Experimental animals and study design

**Figure 1:**
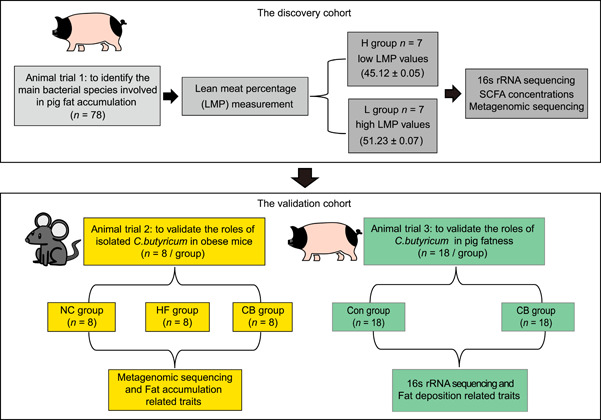
Flow diagram of experimental animals and study design. Three animal trails were included in this study. The first pig trail was used to explore the correlation between the gut microbiome and pig fatness; the second mouse intervention trail was used to validate the roles of isolated *Clostridium butyricum* in fat accumulation in high‐fat (HF)‐diet‐induced obese mice; the third pig intervention trail was used to validate roles of *C. butyricum* in pig fatness. NC, normal chow.

Three animal trails were used in this study (Figure 1). Briefly, the first animal trail comprised 78 male Jinhua pigs, we further chose 14 pigs with extreme phenotype values for 16s rRNA sequencing and metagenomic sequencing to identify the main bacterial species involved in regulating pig fat accumulation. The validation cohort comprised of mouse and pig intervention trails. For mouse trail, 24 male C57BL/6J mice were used to validate the roles of isolated *C. butyricum* in fat accumulation in high‐fat (HF)‐diet‐induced obese mice, mice fed with normal chow (NC) diet was used as a negative control group (Con group), mice fed with HF diet was used as a positive control group (HF group), and mice fed with HF diet and oral gavage with isolated *C. butyricum* was used as validation group (HF_CB group). Furthermore, pig intervention study comprised 36 Jinhua pigs were used to final validate the roles of *C. butyricum* in pig fatness. Jinhua pigs were given ad libitum access to water and normal feed were used as a normal group (Con group) and pigs administered *C. butyricum* combined with diets were used as validation group (CB group).

### The fatness phenotypes of pigs

We used the lean meat percentage (LMP) as an index to divide 14 pigs into high (H) and low (L) fatness groups (Figure S1A,B). No significant differences in body weight were found between the groups (Figure S1C). In comparison with those in the H group, pigs in the L group had an almost 21% lower backfat thickness (Figure S1D) and a 23% decrease in liver fat (Figure S1E). Additionally, the thickness of the ribeye area in low‐fatness pigs was significantly higher than that in high‐fatness pigs, with an average increase of 9.7% (Figure S1F). Furthermore, we detected lipid metabolism‐related genes in the subcutaneous adipose tissue of pigs. The levels of fatty acid synthase (*Fasn*), sterol regulatory element binding protein 1c (*Srebp‐1c*), and peroxisome proliferator‐activated receptor γ (*Pparγ*) were higher in the H group, while *Pparα* and carnitine palmtoyltransferase‐1 (*Cpt‐1*) showed slight increases in the L group (Figure S1G).

### The gut microbiota composition along the GI tract of pigs

**Figure 2:**
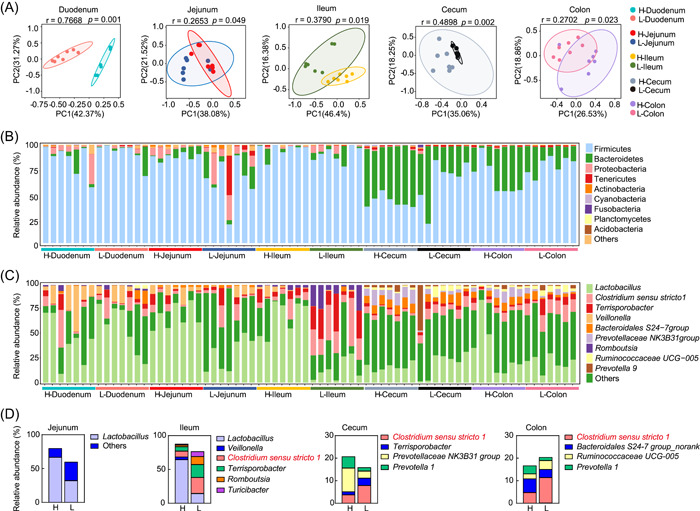
Bacterial 16S rRNA gene sequencing‐based microbiota analysis shows the differences in the gut microbiota in the five different locations along the gastrointestinal tract. (A) Principal component analysis based on the microbiota communities of the samples among different intestine segments. (B) The relative abundance of microbial composition at the phylum level. (C) The relative abundance of microbial composition at the genus level. (D) The significant enrichment of specific genera in the ileum, cecum, and colon, and *Clostridium sensu stricto* 1 (highlighted in red) is mostly found in the low (L) fatness group.

Principal component analysis (PCA) results showed significant differences in bacterial communities between the two groups of pigs in different intestinal segments along the GI tract (Figure 2A). In the duodenum, jejunum, and ileum, the phyla Firmicutes, Proteobacteria, and Bacteroidetes were dominant, making up more than 70%, 10%, and approximately 10% of the microbiota, respectively (Figure 2B). On the other hand, Firmicutes and Bacteroidetes were the most abundant phyla, with a combined average abundance of over 90% in the colon and cecum (Figure 2B). Interestingly, the relative abundance of Firmicutes was significantly increased in the jejunum of the H group (Figure 2B). In addition, the levels of Proteobacteria and Tenericutes were higher in the duodenum of Jinhua pigs with higher fat deposition (i.e., H group), while Cyanobacteria were markedly reduced in the cecum of the L group (Figure 2B). At the genus level, *Lactobacillus*, *Veillonella*, and *Terrisporobacter* were more abundant in areas of the small intestine, such as the duodenum and jejunum, while *Bacteroidales S24‐7 group* and *Prevotella 9* were more enriched in the colon and cecum sections (Figure 2C).

Linear discriminant analysis (LDA) effect size (LEfSe) analysis was further conducted to determine the bacteria that were significantly different between the two groups. Consistent with the above results, the phylum Proteobacteria was more enriched in the small intestines of the H group, while the genus *Clostridium sensu stricto* 1 was enriched in the L group. Moreover, butyric acid‐producing bacterial strains, which belong to the genera *Clostridium* and *Butyrivibrio*, were widely distributed in the low‐fatness pig colon (Figures 2D and Figure S2).

### Association of gut microbiota and short‐chain fatty acids (SCFAs) with fatness in pigs

Given the differences in the gut microbiome along the GI tract observed in Jinhua pigs with different fat deposition, we next examined the SCFA levels in all intestinal segments. As shown in Figure S3, propionate was increased in the ileum of the L group (Figure S3B). Additionally, butyrate was higher in the cecum of the L group (Figure S3E). Greater concentrations of acetate, propionate, butyrate, and isobutyrate were found in the colon of the L group (Figure S3D). The correlation analysis between the top 10 bacterial genera and SCFA levels in the different intestinal segments suggested that microbiome profile differences may contribute to SCFA production (Figure S4).

Next, the results of the PICRUSt2 functional profiles provide a comprehensive view of the diverse functions observed in different intestinal segments (Figure S5). Moreover, the abundance of SCFA production‐related genes, including butyrate kinase (*buk*), phosphate butyryltransferase (*ptb*), d‐lactate dehydrogenase (*ldhA*), propionate kinase (*tdcD*), and phosphotransacetylase (*eutD*), was further compared within the two groups (Figure S6). No statistically significant distinctions emerged in the duodenum, jejunum, or cecum (Figure S6A–D). However, a notable difference emerged in the levels of *buk* and *ptb* in the L group, particularly within the ileum and colon (Figure S6C and Figure 6E).

### Metagenome‐assembled bacterial genomes

**Figure 3:**
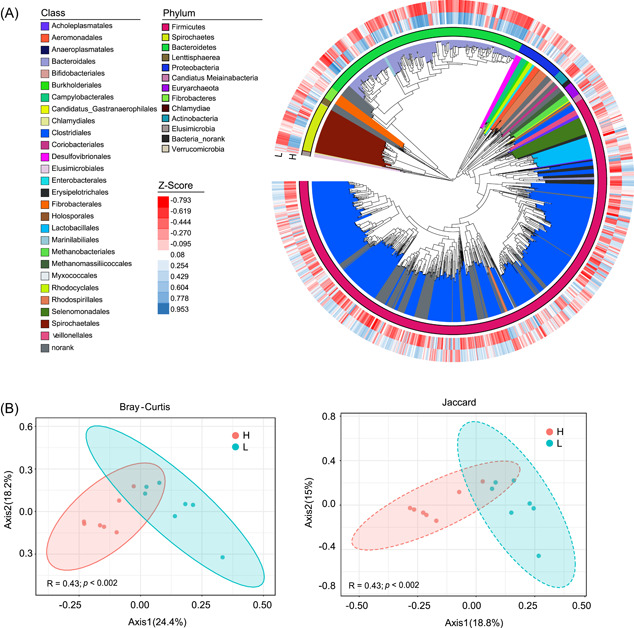
Metagenomic analysis of the colonic microbiome in Jinhua pigs with high fatness (H) and low fatness (L) group (*n* = 7). (A) The reconstruction of 1288 metagenome‐assembled genomes (MAGs) from these pigs. Different colors of the inner circle (i.e., the branches of the phylogenetic tree) indicate the microbial classification at the class level. the different colors of the outer circle represent the phylum‐level classification of these MAGs. The two circles of the heatmap represent the differential representation of these MAGs between the H and L fatness groups (red indicates low abundance. blue indicates high abundance). (B) The principal coordinate analysis plots based on Bray–Curtis and Jaccard distances among the groups. Significant differences in both microbial structure (based on Bray–Curtis distance, analysis of similarity [ANOSIM] *R* = 0.43, *p* < 0.002) and membership (based on Jaccard distance, ANOSIM *r* = 0.29, *p* = 0.013) between the H and L fatness groups were observed.

Reconstruction of bacterial genomes from metagenomic sequence data generated a total of 1288 genomic metagenome‐assembled genomes (MAGs) from these 14 pigs. Figure 3A shows the taxonomic identification of the 1288 MAGs. Among these, 782 MAGs were identified as Firmicutes, 291 as Bacteroidetes, and 68 as Spirochaetes. Furthermore, 64 MAGs were identified as Proteobacteria, 18 as Euryarchaeota, and only 16 MAGs were assigned to Actinobacteria (Figure 3A). The relative abundances of bacteria in the H and L groups were visualized by a heatmap (Figure 3A). The MAGs enriched in the L group were mostly assigned to Firmicutes; conversely, the MAGs identified as Bacteroidetes were more abundant in the H group (Figure 3A). Significant differences in the MAG structure and membership in the two groups were also observed on the principal coordinate analysis (PCoA) plots based on Bray‒Curtis (*r* = 0.43, *p* < 0.002) and Jaccard distances (*r* = 0.29, *p* = 0.013; Figure 3B).

### Functional profiling of the gut microbiome related to fatness based on metagenomic sequencing

The Shannon index based on the level 3 functional pathway indicated a higher functional richness in the L group (Figure S7A). The PCA plot displayed clear distinctions in metabolic potential among the two groups (Figure S7B). Major classes, such as metabolism, genetic information processing, environmental information processing, and cellular processes, were found in both groups (Figure S7C). Moreover, the subclass of each level 1 class is shown in Figure S7D.

**Figure 4:**
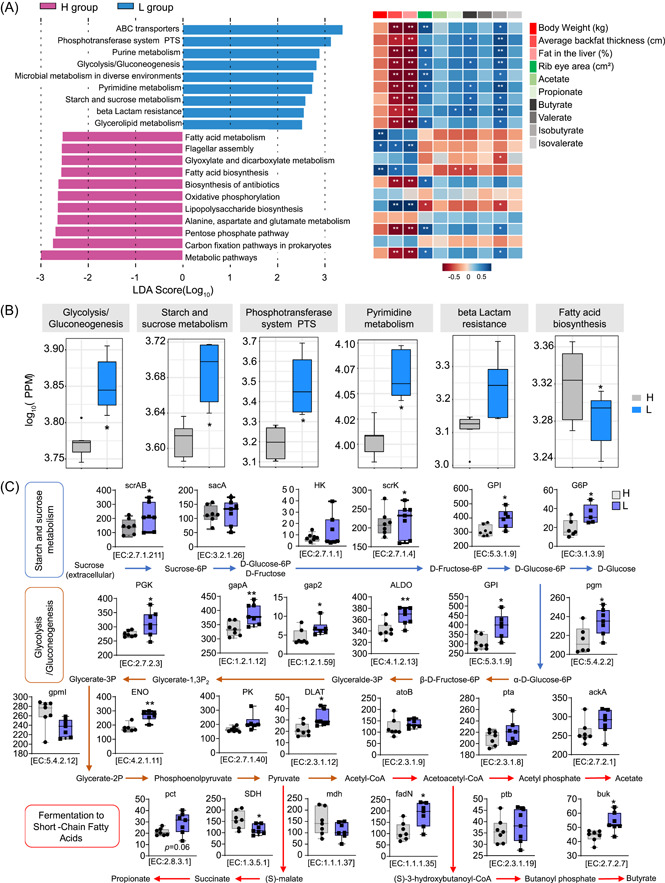
Metagenomic analysis of the differences in metabolic pathways in high fatness (H) and low fatness (L) group (*n* = 7). (A) The differentially represented metabolic pathways at the Kyoto Encyclopedia of Genes and Genomes (KEGG) level 3 through linear discriminant analysis (LDA) effect size determination with LDA value > 2.5. (*p* < 0.01). Spearman's analysis was performed to analyze the correlation between those pathways and related host phenotypes and short‐chain fatty acid levels. Positive correlation is displayed in blue, while negative is marked with red color. (B) The abundance of those specifically identified KEGG functions that showed significant association with butyrate above in Figure 3A. (C) Representative relative enzyme and involved metabolic pathways for butyrate and acetate production by conversion of starch and sucrose in the H and L fatness groups. The pathways were constructed based on the KEGG and MetaCyc database. Arrows indicate the direction of the metabolism reaction. The lines inside the squares represent the median (*n* = 7). Data were presented as mean ± SEM, *p < 0.05, **p < 0.01, ***p < 0.001. ALDO, alditol oxidase; buk, butyrate kinase; DLAT, dihydrolipoamide acetyltransferase; ENO, enolase; fadN, 3‐hydroxyacyl‐CoA dehydrogenase; G6PD, glucose‐6‐phosphate 1‐dehydrogenase; GAPDH, glyceraldehyde 3‐phosphate dehydrogenase; GPI, glucose‐6‐phosphate isomerase; PGK, polyphosphate glucokinase; PGM, phosphoglucomutase; scrAB, sucrose phosphotransferase; scrK, fructokinase; SDH, succinate dehydrogenase.

ABC transporters, the phosphotransferase system, purine metabolism, glycolysis and gluconeogenesis, microbial metabolism in diverse environments, pyrimidine metabolism, starch and sucrose metabolism, beta‐lactam resistance, and glycolipid metabolism were consistently more abundant in the L group (Figure 4A). On the other hand, pigs with high fatness exhibited an increased abundance of microbial profiles involved in fatty acid metabolism, flagellar assembly, glyoxylate and dicarboxylate metabolism, fatty acid biosynthesis, biosynthesis of antibiotics, oxidative phosphorylation, lipopolysaccharide biosynthesis, alanine, aspartate, glutamate metabolism, pentose phosphate pathway, carbon fixation pathways in prokaryotes, and metabolic pathways (Figure 4A).

Additionally, correlation analysis was further conducted to estimate the association between microbiome functional changes with fatness phenotypes and SCFAs in the pigs (Figure 4A). Interestingly, those L group‐enriched pathways were negatively correlated with body weight, average backfat thickness, and liver fat but positively associated with ribeye area and SCFA levels (Figure 4A and Table S1). In contrast, the pathways associated with high fatness exhibited a negative correlation with ribeye area and SCFA levels but showed a significantly positive relationship with body weight, average backfat thickness, and liver fat (Figure 4A). The L group exhibited significantly higher activity in glycolysis/gluconeogenesis, starch and sucrose metabolism, phosphotransferase system, pyrimidine metabolism, and lactam resistance. These pathways were found to be positively correlated with butyrate and isobutyrate levels (Figure 4A,B). Conversely, fatty acid biosynthesis, which increased in the H group, was negatively associated with butyrate and isobutyrate (Figure 4A,B).

Consequently, we compared the abundance of KEGG Orthologs (KO) genes related to the pathways between the H and L metagenomes, including starch and sucrose metabolism, glycolysis/gluconeogenesis, and SCFA fermentation pathways. Figure 4C illustrates the fermentation pathway responsible for metabolizing sucrose into acetate, butyrate, and propionate. The metagenome sequencing data indicated a higher abundance of these genes in the L group, especially sucrose phosphotransferase, fructokinase, glucose‐6‐phosphate isomerase, glucose‐6‐phosphate 1‐dehydrogenase, polyphosphate glucokinase, glyceraldehyde 3‐phosphate dehydrogenase, alditol oxidase, phosphoglucomutase, enolase, dihydrolipoamide acetyltransferase, succinate dehydrogenase, 3‐hydroxyacyl‐CoA dehydrogenase, and *Buk* (Figure 4C and Table S2).

### Comparisons of the CAZymes genes encoded by the gut microbiome in pigs

Various classes of CAZymes, including auxiliary activities (AA), carbohydrate‐binding modules (CBM), glycoside hydrolases (GH), glycosyl transferase (GT), polysaccharide lyase (PL), and S‐layer homology domain (SLH), were identified. As shown in Figure S9A, the number of genes encoding CAZyme classes, including CBM, CE, GT, and especially GH, was higher than that of other CAZymes in the complete metagenomic pool (Figure S8A). Importantly, different bacterial taxa may possess distinct pathways for carbohydrate degradation. For instance, Bacteroidetes predominantly encoded PL enzymes, which are involved in the cleavage of uronic acid‐containing polysaccharide chains. On the other hand, Firmicutes were the primary source of cohesin‐ and dockerin‐encoding genes. CBM, CE, GH, and GT were encoded by various phyla, with Firmicutes and Bacteroidetes being the most prominent contributors (Figure S8A).

**Figure 5:**
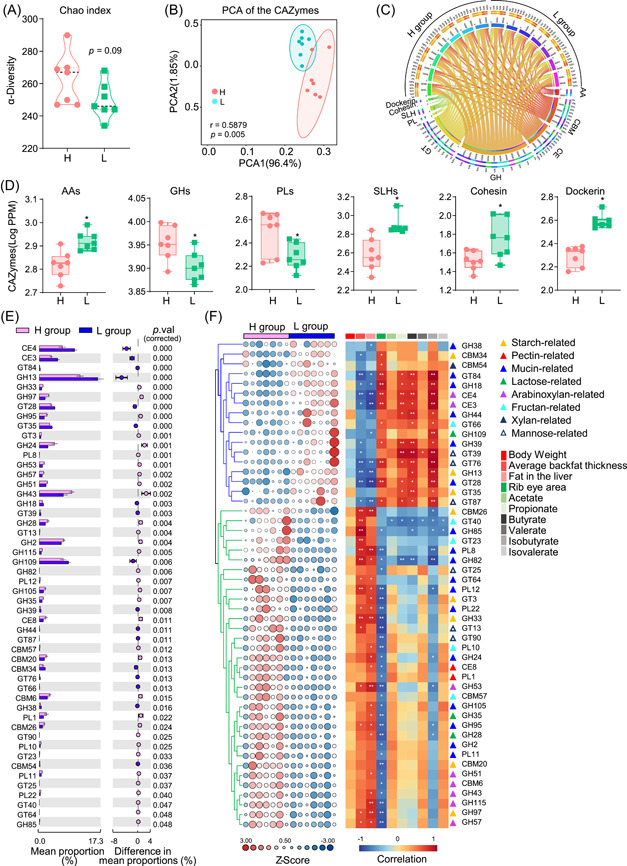
Metagenomic analysis of the differentially represented genes encoding CAZymes between the high fatness (H) and low fatness (L) group (*n* = 7). (A) The CAZymes gene diversity (Chao 1 index) in the two groups. (B) The differences in CAZyme gene profiles between the H and L groups based on the principal‐coordinate analysis using Bray–Curtis distances. (C) The circle circos plot shows the different CAZymes patterns among the H and L groups (*n* = 7). (D) The core CAZyme families that were significantly different in the H and L groups by using STAMP (v2.1.3). (E) The statistically significant CAZymes in the two groups. (F) The correlations between these CAZyme families and phenotypes, including body weight, average backfat thickness, liver fat, ribeye area, and short‐chain fatty acids concentration in the colon. Positive correlation is displayed in red, while negative is marked with blue color. The different triangle color means the responsible for starch‐, pectin‐, mucin‐, lactose‐, arabinoxylan‐, fructan‐, xylan‐, and mannose‐related CAZyme. Data were presented as the mean ± SEM, *p < 0.05, **p < 0.01, ***p < 0.001. AA, auxiliary activities; CBM, carbohydrate‐binding modules; CE, carbohydrate esterase; GH, glycoside hydrolase family; GT, glycosyl transferase; PCA, principal component analysis; PL, polysaccharide lyase; PTS, phosphotransferase system; SLH, S‐layer homology domain.

We proceeded to analyze the differences in CAZymes between the two groups, and interestingly, the L group exhibited a lower diversity of CAZymes than the H group (Figure 5A). PCA further highlighted a significant difference in CAZyme profiles between the two groups (*r* = 0.5879, *p* = 0.005; Figure 5B). When examining the nine CAZyme families, the L group displayed significantly lower abundances of genes encoding GHs and PLs, while exhibiting higher levels of AAs, SLHs, cohesin, and dockerin (Figure 5C,D). Figure S8B depicts the relationships between these carbohydrate‐active enzymes and the top 15 bacterial phyla, including Bacteroidetes, Firmicutes, and Proteobacteria (Figure S8B). The differentially represented CAZymes, including AAs, GHs, PLs, SLHs, cohesin, and dockerin, between the two groups were primarily assigned phylogenetically to Bacteroidetes and Firmicutes at the phylum level (Figure S8C).

### The changes of CAZymes linked to SCFA production and fatness traits in pigs

Moreover, we identified 134 distinct families of GHs, 99 families of GTs, 22 families of PLs, 15 families of CEs, 79 families of associated CBMs, and five families of associated AAs among the unique genes (Table S3). To explore the co‐occurrence patterns of CAZymes, we constructed a network based on strong and significant correlations (Spearman's correlation > 0.6; *p* < 0.01). The results revealed that the CAZyme networks were more complex in pigs with low fatness, with 1617 links in the H group and 3031 links in the L group (Figure S9).

Subsequently, we conducted a more detailed analysis of the statistically significant CAZyme families between the two groups of pigs with different fatness phenotypes (Figure 5E). These CAZyme families were primarily associated with the metabolism of starch, lactose, fructan, mucin, arabinoxylan, mannose, pectin, and xylan. Notably, the CAZymes enriched in the L group showed a positive correlation with SCFA production and ribeye area while exhibiting a negative correlation with average backfat thickness and liver fat (Figure 5E,F). Conversely, the CAZymes associated with the high‐fatness group displayed an inverse relationship with these phenotypes (Figure 5F). These findings strongly suggest that microbiota‐mediated CAZymes play a crucial role in regulating host phenotypes.

### Identifying a significant change of *C*. *butyricum* in pigs with low fatness and its association with CAZymes

**Figure 6:**
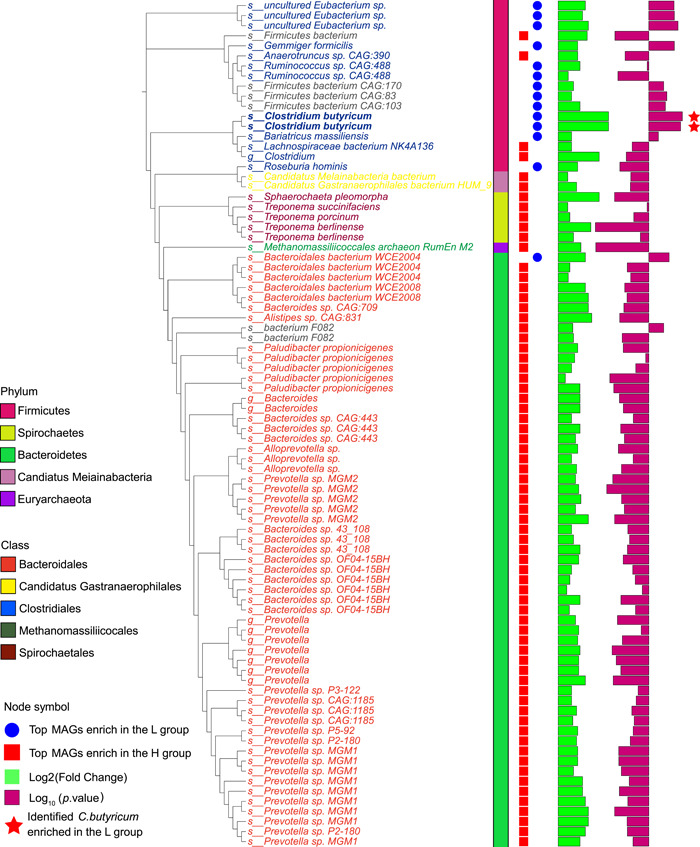
Identifying a significant change of *Clostridium butyricum* in pigs with low fatness. The 84 potential metagenome‐assembled genome (MAG) biomarkers differentially represented between the high (H) and low (L) fatness groups through linear discriminant analysis (LDA) effect size determination with LDA value > 3.5. The MAGs associated with *C. butyricum* was the most significant one enriched in the L fatness group.

Metagenome sequencing showed that more MAGs were enriched in the H group (*n* = 70 MAGs) than in the L group (*n* = 14 MAGs) (Figure 6). Among the differentially enriched MAGs, two MAGs (Bin 314 and Bin 375) associated with *C*. *butyricum* had the largest change in the L group (Figures 6 and Figure S10). Since the most abundant genes encoding GH13 enzymes were enriched in the L group, we further explored the distribution of CAZymes, especially of GH13 in *C. butyricum* genome (Table S12). Phylogenetic assignment revealed that these genes were primarily associated with the Firmicutes phylum, specifically the *Clostridium* genus (Figure S11A,B). Furthermore, specific species, such as *Clostridium* sp. CAG:221, *C. bacterium* Marseille‐P2846, and *C. butyricum*, were identified as contributors to GH13 enrichment in the L group (Figure S11A–C). Within the phylum Firmicutes, these bacteria are known to convert starch through the action of an extracellular *α*‐(1 → 4)‐glucan branching enzyme, which was found to be more abundant in the L group (Figure S11D).

Considering that *C. butyricum* was one of the most significantly enriched MAGs in the L group and exhibited an increase in CAZymes, we formulated the hypothesis that *C. butyricum* plays a crucial role in host fatness. We initially investigated the distribution of CAZymes in *C. butyricum* (Figure S12) and found that 32.38% of the enzymes were dockerin, 25.12% were GHs, 15.39% were CBMs, 12.66% were GTs, 9.74% were CEs, 2.74% were AAs, 1.65% were SLHs and 0.34% were PLs (Figure S12A). Dockerin, CBM50, CE4, GH13, and GH1 were the most abundant CAZyme families found in the *C. butyricum* genome (Figure S12B). Additionally, sequences assigned to CEs, GTs, CBMs, and GHs were distributed along 4, 10, 9, and 16 families in the *C. butyricum* genome (Figure S12C).

### Isolated *C*. *butyricum* alleviated fat accumulation and increased the enrichment of GH13 in obese mice

**Figure 7:**
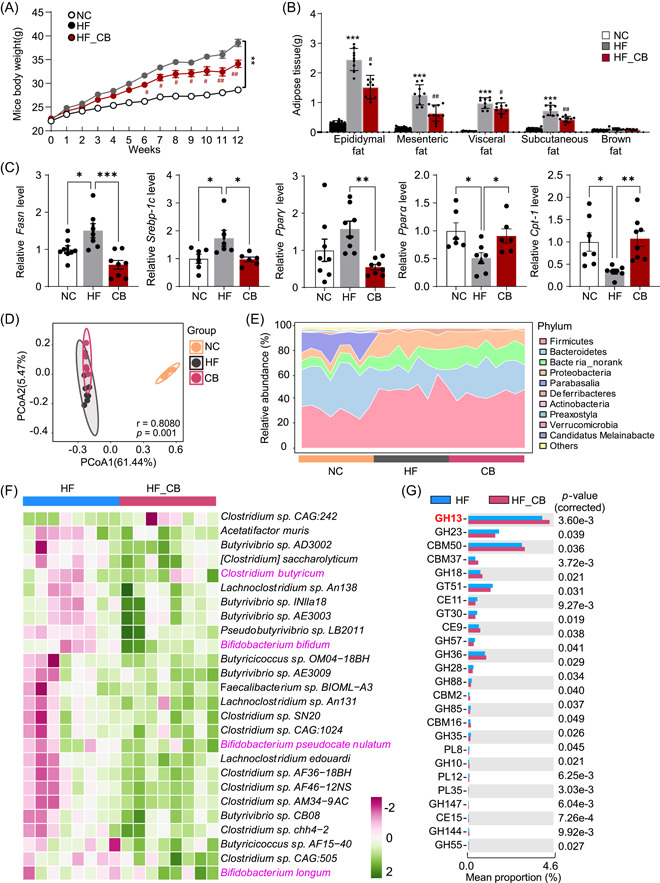
Effect of *Clostridium butyricum*, isolated from Jinhua pigs on the gut microbiome profiles and fat accumulation in high‐fat (HF) diet‐induced obese mice. (A) The mice body weight during the 12 weeks of *C. butyricum* treatment (*n* = 8). (B) The adipose tissue of epididymal, mesenteric, visceral, subcutaneous, and brown fat. (C) The expression of lipogenesis and lipolysis‐related genes in subcutaneous adipose tissue. (D) Principal‐coordinate analysis shows the microbiota community changes between the normal chow, high‐fat (HF), and HF_CB groups. (E) Relative abundance of microbiota at the phylum level among these three groups. (F) Heatmap shows the abundance of identified short‐chain fatty acid‐producing bacteria species, such as *C. butyricum* in HF and HF_CB groups. (G) Differences in CAZymes between the HF and HF_CB groups. Data were presented as the means ± SEM, *p < 0.05, **p < 0.01 versus NC. ^#^ *p* < 0.05, ^##^ *p* < 0.01 versus HF. *Cpt‐1a*, carnitine palmitoyltransferase 1A; *Fasn*, fatty acid synthase; *Ppara*, peroxisome proliferator‐activated receptor alpha; *Pparg*, peroxisome proliferator‐activated receptor gamma; *Srebp‐1c*, sterol regulatory element binding protein‐1.

We next assessed the potential causal relationship between *C. butyricum* isolated from experimental pigs with fat accumulation and CAZymes in HF diet‐induced obese mice. After 12 weeks, quantitative polymerase chain reaction (PCR) analysis of fecal DNA confirmed the successful colonization of *C. butyricum* in the guts of treated mouse, *C. butyricum* led to a reduction in diet‐induced body weight gain compared to mice on an HF diet (Figure 7A and Figure S13). Furthermore, *C. butyricum* significantly decreased body fat accumulation, including epididymal, mesenteric, visceral, and subcutaneous fat, indicating that the decrease in weight gain observed in *C. butyricum*‐treated mice was primarily due to a significant decrease in fat mass (Figure 7B). Following intervention with *C. butyricum* in HF mice, we conducted reverse transcription (RT)‐PCR analyses to investigate the expression of genes associated with lipogenesis and lipolysis in subcutaneous adipose tissue. Remarkably, the messenger RNA (mRNA) levels of lipogenesis genes, notably *Srebp‐1c*, *Pparγ*, and *Fasn*, exhibited a significant decrease in adipose tissue after *C. butyricum* intervention (Figure 7C). In the context of lipolysis‐related genes, there was a substantial elevation in the mRNA levels of *Ppara* and *Cpt‐1a* within the CB group (Figure 7C).

The PCoA results demonstrated that the β‐diversity value could effectively differentiate between lean mice fed a NC diet and obese mice fed an HF diet (Figure 7D). Notably, treatment with *C. butyricum* resulted in even greater discrimination compared to obese mice (*r* = 0.8080, *p* = 0.001; Figure 7D). At the phylum level, the overall composition of the gut microbiota revealed an increase in Firmicutes and a decrease in Bacteroidetes in HF diet‐induced obese mice, which were restored by *C. butyricum* treatment (Figure 7D). Importantly, the abundance of *C. butyricum* increased after *C. butyricum* treatment (Figure 7E). Additionally, *C. butyricum* treatment led to an increase in SCFA‐producing bacterial species, including *Bifidobacterium bifidum*, *Bifidobacterium pseudocatenulatum*, and *Bifidobacterium longum* (Figure 7E). The bacterial CAZyme GH13 was enhanced by *C. butyricum* treatment, resembling the low‐fat group in previous experiments (Figure 7F). Overall, the enrichment of GH13 enzymes, specifically in relation to *C. butyricum*, suggests a potential relationship between GH13 enzymes and fatness regulation.

### *C. butyricum* treatment alleviated fat accumulation in Jinhua pigs

**Figure 8:**
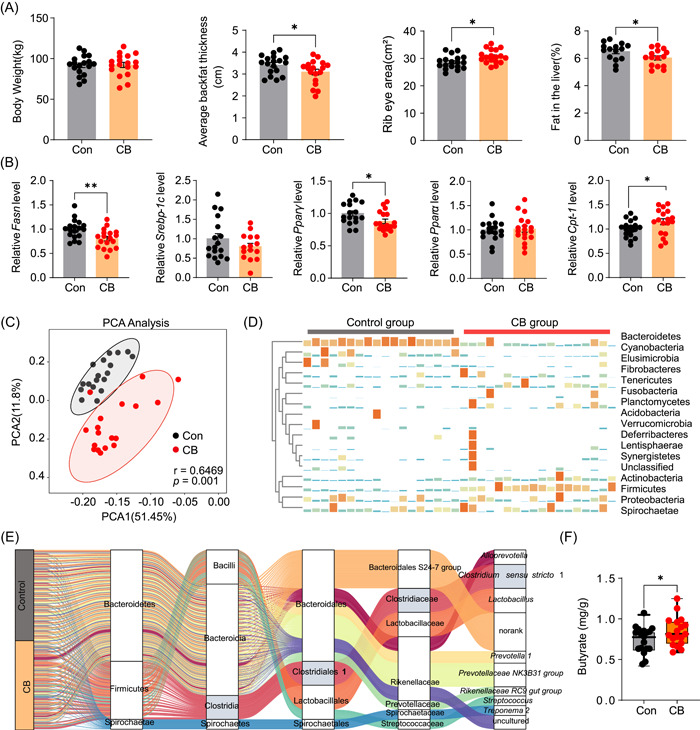
*Clostridium butyricum* treatment alleviated fat accumulation in Jinhua pigs. (A) The phenotypes, including body weight, average backfat thickness, ribeye area, and liver fat in the control and CB groups. (B) The expression of lipogenesis and lipolysis‐related genes in subcutaneous adipose tissue of pigs. (C) Principal component analysis shows colonic microbiota profiles in the two groups. (D) The relative abundance of phyla in the two groups. (E) Sankey diagram showing the relative contribution of the top 10 Operational Taxonomic Units' to the taxonomic diversity. From left to right, names refer to phyla, class, orders, family, and genus. (F) Short‐chain fatty acid levels in the colon of the two groups. Data were presented as the means ± SEM, *p < 0.05, **p < 0.01 versus Con.

Since bacterial species directly isolated from pigs need to be verified for safety before they can be used in swine production, we next examined the role of commercialized *C. butyricum* isolated from pigs on the fatness phenotype of Jinhua pigs. Compared with the control group, pigs fed *C. butyricum* had a significant increase in ribeye area and lower average backfat thickness and liver fat (Figure 8A). RT‐PCR results further showed that *Fasn* and *Pparγ* were significantly increased with *C. butyricum* treatment, while the mRNA level of *Cpt‐1* was downregulated in the CB group (Figure 8B). In addition, the PCA plot showed a clear separation of the gut microbiota between the control and CB groups (Figure 8C). At the phylum level, *C. butyricum* treatment significantly increased the relative abundances of *Actinobacteria*, *Firmicutes*, *Proteobacteria*, and *Spirochaeta* (Figure 8D). On the other hand, *Bacteroidetes* were reduced in the *C. butyricum* groups (Figure 8D). The Sankey plot displays the composition of the microbiota at the phylum to genus levels (Figure 8E). Importantly, the abundance of *C. sensu stricto* 1 was significantly increased after *C. butyricum* treatments, which was similar to the trends in the lower‐fatness Jinhua pigs (Figure 8E). More importantly, the CB group pigs had a higher butyrate concentration in the colon than the control group (Figure 8F and Figure S14).

## DISCUSSION

The gut microbiota has been reported to be an environmental factor that regulates fat storage phenotypes, digestibility, and growth performance [19, 20]. In our previous studies, we compared the biogeography of gut microbiotas collected from different segments of the intestinal tract of Jinhua and Danish Landrace pigs, which are the two swine breeds with distinct fat deposition phenotypes, and revealed the correlation of gut microbiota with adipogenicity [15, 16]. Herein, PCA plots showed distinct clusters completely separating the microbiomes among different intestinal segments between the two groups, suggesting dramatic changes in community structure and composition in the different intestinal segments of Jinhua pigs with distinct fatness phenotypes. Moreover, the abundance of the *Firmicutes*, *Bacteroidetes*, and *Proteobacteria* phyla accounted for a large proportion of the gut microbiota among all intestinal segments in pigs in our study, which was in line with previous studies [21].

SCFAs, such as butyrate, play a pivotal role in connecting microbiota functions to diverse biological effects [22, 23, 24, 25]. Specifically, butyrate represents a significant energy source for the colonic epithelium [26, 27]. Previous studies conducted in pigs have demonstrated that dietary supplementation with sodium butyrate following weaning enhances growth performance during the initial and subsequent postnatal months [28, 29]. Furthermore, oral administration of SCFAs has been shown to attenuate fat deposition in weaned pigs by reducing lipogenesis and enhancing lipolysis [30]. Moreover, higher SCFA levels were detected in this study, especially butyrate, in pigs with less fat accumulation, supporting the important role of SCFAs in regulating fat storage. Notably, the two genes (*ptb* and *buk*) of the *buk* pathway enzymes [31, 32] were identified as higher in the colon of l‐fatness pigs, which not only supports the key roles of *buk* and *ptb* in butyrate formation but also explains why we observed a very profound increase in butyrate levels in the colon of Jinhua pigs. Furthermore, the correlation between microbiota abundance and SCFA levels revealed a stronger correlation of butyrate with *C. sensu stricto* 1, suggesting that the butyrate‐producing bacteria enriched in the L group may affect host fat deposition.

Our study highlighted *C. butyricum* as the predominant bacterium in pigs with lower fatness. *C. butyricum*, a butyric acid‐producing gram‐positive obligate anaerobic bacterium belonging to *Clostridium* cluster I, is commonly found in the intestines of animals and humans and utilized as a probiotic [33, 34, 35, 36]. Beyond the positive effects on SCFA production, numerous reports have demonstrated the positive effects of *C. butyricum* on growth performance, nutrient utilization, and gut health in animals [37, 38, 39, 40]. Additionally, *C. butyricum* was reported to regulate lipid metabolism in animals, such as decreasing TG synthesis, accelerating fatty acid oxidation, and shaping the gut microbiota and bile acid profile [41, 42]. These findings were further supported by mouse intervention herein, which demonstrated the ability of isolated *C. butyricum* to alleviate fat accumulation induced by HF diets. The reduction in fat deposition associated with *C. butyricum* intervention might be attributed, at least in part, to the concurrent reduction in lipogenesis and enhancement of β‐oxidation, as evidenced by a pronounced increase in *Ppara* and a substantial elevation in the mRNA level of *Cpt‐1a*. These findings suggest that *C. butyricum* may be a core species within the microbiota involved in fat deposition in pigs.

The colonic microbiome encodes many CAZymes to degrade carbohydrates beyond the capabilities of the host [43, 44]. A previous study showed that significant differences in the abundance of CAZymes were observed between fat and lean pigs, with lean pigs exhibiting a higher enrichment of CAZymes involved in galactose, xylan, and mannose metabolism [45]. In our study, CAZyme families encompass various molecular enzyme functions, and the diversity of CAZymes in the two groups reflects the varying capacity for carbohydrate degradation in pigs. Correlation analysis between the CAZymes and fatness‐related traits further underscores the contribution of CAZymes to host phenotypes. Another noteworthy discovery in our study is the comparison of CAZyme association networks between pigs with high and low fatness, which revealed a more intricate network in pigs with lower fatness. This complexity may facilitate improved carbohydrate degradation and energy metabolism.

An increasing amount of evidence demonstrates a tight link between *C*. *butyricum* and enzymes involved in carbohydrate degradation [46, 47]. We identified a variety of CAZyme families in *C*. *butyricum* strains. For example, *C. butyricum* constituted 8.15% of sequences belonging to CBM50, which is closely linked to several GHs capable of breaking down chitin or peptidoglycan [48]. Another prominent CAZyme group identified in *C. butyricum* strains was the CE4 family, which plays a crucial role in the deacetylation of xylans and xylooligosaccharides [49]. The prevalence of CE4 enzymes in *C. butyricum* indicates the strain's ability to efficiently modify and utilize these complex carbohydrates. Furthermore, our analysis revealed a significant abundance of glycosyl hydrolase GH13, particularly in the L group. GH13 belongs to the α‐amylase family and serves as the principal enzyme responsible for starch degradation [50]. The increased presence of GH13 in pigs with lower fat deposition suggests that these animals may exhibit enhanced accessibility to insoluble starch, leading to improved utilization and potentially contributing to their leaner phenotype.

These findings underscore the intricate relationship between *C. butyricum* and a wide array of CAZyme families involved in carbohydrate degradation. These results provide valuable insights into the mechanisms underlying the strain's ability to efficiently breakdown and utilize various complex carbohydrates, such as chitin, peptidoglycan, xylans, xylooligosaccharides, and starch. Further research in this area could uncover novel applications for *C. butyricum* in enhancing carbohydrate utilization and promoting animal health. However, importantly, this study has a limitation in terms of the relatively small sample size. Therefore, it is imperative to conduct further investigations with a larger sample size and employ deeper sequencing techniques to validate and expand upon these findings.

## CONCLUSION

In summary, our study reveals that the gut microbiome composition and SCFA production vary across different intestinal segments in Jinhua pigs with distinct fatness phenotypes, thereby contributing to the host's fat storage phenotype. Furthermore, the dissimilarities observed in genes related to CAZymes suggest that the gut microbiomes of pigs possess different abilities to efficiently utilize dietary carbohydrates. The increased presence of GH13 in pigs with lower deposition suggests that these animals may exhibit enhanced accessibility to insoluble starch, leading to improved utilization and potentially contributing to their leaner phenotype. Importantly, *C. butyricum* may represent one of the core species within the microbiota that contributes to fat deposition in pigs. This study outlines gut microbiota, especially probiotic mechanisms of regulating animal fat deposition, providing a foundation of potential interventions for human metabolic disorders.

## METHODS

### Animal trial 1: To explore the correlation between the gut microbiome and pig fatness

During the study, 78 male Jinhua pigs (180 days old) were raised in the environmentally controlled facility, where 9–10 pigs were housed per pen at the Jinhua Academy of Agricultural Sciences Experimental Farm. All experimental pigs were raised under similar feeding and management conditions a standard commercial corn–soybean diet. During the whole experimental period, pigs were allowed to access water ad libitum. The commercial formula feed contained 60.9% corn, 20.5% soybeans, and 13.5% wheat bran, including 13.21 MJ kg^−^^1^ digestible energy and 15.15% crude protein. The main nutrient components of the diets are listed in Table S5.

All animals were killed after 270 days of age. Backfat thickness was measured in the middle of the last third and fourth ribs. Ribeye area was measured between the 10th and 11th ribs on pork carcasses. LMP was calculated based on fat and muscle thickness data measured by Autofom ultrasound device (Frontmatec) [40]. LMP was used as an index to assess the role of the gut microbiome in porcine fatness in animal trail 1 (Figure 1). The phenotypic values of LMP obey a normal distribution (Figure S1A). Then, we chose 14 fecal samples with extreme phenotype values for a further 16s rRNA and metagenomic sequencing, including seven samples with high fatness (H group) and low LMP values (45.12 ± 0.05); seven samples with low fatness (L group) and high LMP values (51.23 ± 0.07) (Figure 1).

### Animal trial 2: To validate the roles of isolated *C. butyricum* in fat accumulation in HF‐diet‐induced obese mice

Mice were kept in a temperature‐controlled room (22 ± 2°C) under a 12 h dark‐light cycle under specific‐pathogen‐free conditions and provided with ad libitum access to water and chow (four mice per cage). After 1 week of acclimatization, 8‐week‐old male C57BL/6J mice (China National Laboratory Animal Resource Center) were divided into three groups (*n* = 8 each group) and fed for 12 weeks as follows: NC group (P1101F‐25, Salcom Co. Ltd.) was used as a control; HF diet (HF group, 60% fat, Research Diets Inc., D12492); HF with *C*. *butyricum* (HF_CB group, 10^8^ CFU, 200 μL) was administered by daily gavage according to our previous study [15]. Food and water were available ad libitum. After 12 weeks, adipose tissue samples of epididymal, mesenteric, visceral, subcutaneous, and brown fat were isolated and weighed for all groups.

### Animal trial 3: To validate the roles of *C. butyricum* in pig fatness

Since bacteria species directly isolated from pigs need to be verified for safety before they can be used in swine production, we next examined the role of commercialized *C. butyricum* isolated from pigs on the fatness phenotype of Jinhua pigs. *C. butyricum* isolated from pigs was provided by Huijia Biological Technology Co. Ltd. A total of 36 Jinhua pigs (180 days of age) were allocated to two groups according to their body weight (*n* = 18/each group): (1) Control group: Jinhua pigs were given ad libitum access to water and feed (Con group); (2) CB group: Jinhua pigs were administered 10^10^ CFU/kg body weight combined with diets and given ad libitum access to water and feed for 12 weeks (CB group) (Figure 1). At 270 days of age, all animals were killed. Backfat thickness and eye muscle area of pigs were calculated, and liver and colon contents were collected and analyzed as described previously [15]. All experimental pigs were raised under the same feeding and management conditions. The 78 purebred Jinhua pigs for the first animal trial and the 36 purebred Jinhua pigs for this animal trial were offspring from the same 12 breeding sows with the same boar to make sure the piglets were at least half‐siblings.

### Metagenomic sequencing analysis

Metagenomic library with an insert size of 350 bp was constructed from high‐quality DNA extracted from each of the 14 samples (the colonic contents of H group and L group for animal trial 1) and 16 samples from obese mice (the colonic contents of HF and HF_CB group for animal trial 2) using the TruSeq DNA PCR‐Free Library Preparation Kit (Illumina) following the manufacturer's instructions and sequenced on a Novaseq. 6000 platform at Mingke Biotechnology Co. Ltd.

Raw reads were filtered using Trimmomatic (v0.32) to remove (i) all reads less than 50 bp in length, (ii) reads with degenerated bases (N's), and (iii) all duplicates defined as sequences whose initial 20 nucleotides were identical and shared an overall identity similarity of >97% throughout the length of the shortest read. Megahit (v1.2.9) was used to assemble these clean reads into contigs, and Prodigal was used for gene prediction from the contigs and obtaining the gene profiles per metagenome [51, 52, 53]. Next, the nonredundant gene set was clustered by CD‐HIT at 95% identity and 90% coverage [54]. Based on these gene profiles, Salmon was used to map the clean reads (keeping only the reads that theoretically belong to Prokaryotes) per metagenome to the clean nonredundant gene profile and obtained the transcripts per million reads abundance of these nonredundant gene profiles in each metagenome [55]. Finally, we blasted these genes against the NR database in NCBI using diamond and gained the putative taxon assignments of these genes per metagenome.

Binning was performed with Metabat2 [56]. Use BWA software to map clean reads to the final nonredundant high‐quality MAG, and calculate the relative abundance and expression of MAG. Quality assessment of each MAGs recovered was conducted using CheckM (v1.0.12). The merge method of CheckM was used to combine bins from the same microbial population to increase completeness (≥80%) and reduce contamination (≤10%). After a two‐step construction of bins, the 1288 MAGs (completeness ≥ 80% and contamination ≤ 10%) were regarded as high‐quality assembled genomes and selected for further analyses (Tables S6–8). Fast whole‐genome average nucleotide identity (ANI) was used to calculate the ANI between each MAG (ANI ≥ 95%) [57]. Taxonomic annotation of MAGs was performed using the Genome Taxonomy Database Toolkit (v2.1.0) [58]. The phylogenetic tree was constructed by PhyloPhlAn (v3.0.51) and visualized by iTOL (v5.6.2).

### Functional annotation

Functional annotation of the “nonredundant” gene catalog was performed using BLASTP against the KEGG database (2021.07; https://www.genome.jp/kegg/) to obtain KO with an *e* value of 10^−5^ [59]. Then, we used custom Perl scripts to gain the abundance part per million (PPM, one KO pathway‐assigned sequence per million sequences) of KO pathways for each metagenome. LEfSe was used to identify the significant difference in the abundance of KEGG pathways between high and low fatness pigs based on the LDA > 3, *p* < 0.05. CAZymes were annotated by using HMMER (v.3.2.1) to match protein sequences to entries in the hidden Markov model libraries of CAZyme families downloaded from the CAZyme database (v.7; http://www.cazy.org/) [60, 61] (Table S4). Circos was used to visualize the contribution of bacteria taxon regarding the CAZyme families based on the PPM (one CAZyme‐like sequence per million sequences) of bacterial genera for the annotated CAZyme families. The correlation analysis was performed by Spearman's correlation analysis. Gephi (v.0.9.1) was utilized to visualize the network of correlations between CAZyme families. The significance of CAZyme families between the high and low fatness groups was determined using Welch's *t* test and Benjamini–Hochberg false discovery rate correction available in Statistical Analysis of Metagenomic Profiles software [62]. The heatmap was generated using TBtools software (a Toolkit for Biologists integrating various biological data‐handling tools) [63].

### Statistical analysis

Data were represented as means ± SEM. Student's *t* tests or one‐way analysis of variance were performed to analyze the phenotype differences by using the Prism 9.0 program (GraphPad Software). PCoA plot based on the Bray–Curtis ordination was constructed to assess dissimilarity among these groups. Analysis of similarity (ANOSIM) and permutational multivariate analysis of variance were performed to test group‐level differences using the R *vegan* package (v2.5.4). The adjusted *p* < 0.05 indicates statistical significance. Bioinformatic analysis was performed using the OmicStudio tools at https://www.omicstudio.cn/tool, and online software TUTU analysis platform (https://www.cloudtutu.com/).

## AUTHOR CONTRIBUTIONS

Lingyan Ma, Shiyu Tao, and Tongxing Song performed the experiments, analyzed data, prepared figures, and wrote the manuscript. Wentao Lyu, Ying Li, Wen Wang, and Qicheng Shen participated in experiments. Yan Ni and Jiang Zhu collected samples. Jiangchao Zhao, Hua Yang, and Yingping Xiao contributed to the study concept, design, and revised the manuscript. All authors have read the final manuscript and approved it for publication.

## CONFLICT OF INTEREST STATEMENT

The authors declare no conflict of interest.

## ETHICS STATEMENT

The ethics application (2019ZAASLA96) was approved by the Institutional Animal Care and Use Committee of the Zhejiang Academy of Agricultural Sciences.

## Supporting information


**Figure S1**: The fatness phenotypes of pigs.
**Figure S2**: The differentially represented bacterial communities through Linear Discriminant Analysis (LDA) Effect Size determination with LDA value > 2.5 along the GI‐tract (*p* < 0.01).
**Figure S3**: The short‐chain fatty acids (SCFAs) concentrations varied in different intestine segments of Jinhua pigs.
**Figure S4**: The correlation analysis between identified top10 genus bacteria with the short‐chain fatty acids (SCFAs) levels in the different intestine segments, including duodenum, jejunum, ileum, colon, and cecum.
**Figure S5**: The differentially represented metabolic pathways at the Kyoto Encyclopedia of Genes and Genomes (KEGG) level 3 through Linear Discriminant Analysis (LDA) Effect Size determination with LDA value > 2.5 and *p* < 0.01 along the GI‐tract based on the PICRUSt2.
**Figure S6**: Short‐chain fatty acids (SCFAs) producing related gene expression along the GI‐tract.
**Figure S7**: The functional microbiome profiles of gut microbiome between high and low fatness pigs through functional annotation of metagenome with the Kyoto Encyclopedia of Genes and Genomes (KEGG) database.
**Figure S8**: The Carbohydrate‐Active enZymes (CAZymes) distribution in groups.
**Figure S9**: Network of co‐occurring Carbohydrate‐Active enZymes (CAZymes) based on correlation analysis in the H (*n* = 7, left panel) and L (*n* = 7, right panel) fatness pigs.
**Figure S10**: Significant changes of bins between two groups.
**Figure S11**: Phylogenetic distribution of sequences in glycoside hydrolase family 13 (GH13) assigned to the identified bacteria.
**Figure S12**: Carbohydrate‐active enzymes distribution in *Clostridium butyricum*.
**Figure S13**: qPCR confirmed the successful colonization of *Clostridium butyricum* in obese mice.
**Figure S14:** Short‐chain fatty acids (SCFAs) levels in the colon of the two groups.

**Table S1:** Correlation between KEGG function and phenotype and SCFAs of pigs.
**Table S2:** The abundance of identified function in groups.
**Table S3:** CAZymes coding numbers in 1288 genomes.
**Table S4:** Detailed information of assembled genomes.
**Table S5:** Composition and nutrient levels of basal diets.
**Table S6:** Statistics of raw data.
**Table S7:** Statistics of clean data (reads).
**Table S8:** Statistics of clean data (contigs).
**Table S9:** Primer sequence used for RT‐PCR.
**Table S10:** Primer sequence used for qPCR.
**Table S11:** Annotation of CAZymes in high‐quality Bin 314.
**Table S12:** Annotation of CAZymes in high‐quality Bin 375.

## Data Availability

(16S rRNA and metagenomic sequencing data were submitted to the NCBI Sequence Read Archive (SRA) database under the study accession numbers PRJNA765142 https://www.ncbi.nlm.nih.gov/bioproject/?term=PRJNA765142), PRJNA766255 (https://www.ncbi.nlm.nih.gov/bioproject/?term=PRJNA766255), and PRJNA761907 (https://www.ncbi.nlm.nih.gov/bioproject/?term=PRJNA761907). Supporting Information (methods, figures, tables, scripts, graphical abstract, slides, videos, Chinese translated version, and update materials) may be found in the online DOI or iMeta Science http://www.imeta.science/.
