# Supplementary material for: Clostridium butyricum and carbohydrate active enzymes contribute to the reduced fat deposition in pigs
Source: Imeta. 2024 Jan 3;3(1):e160. doi: 10.1002/imt2.160 (PMC10989082; doi:10.1002/imt2.160)
Supplement: Supplementary file 1 — Figure S1: The fatness phenotypes of pigs. Figure S2: The differentially represented bacterial communities through Linear Discriminant Analysis (LDA) Effect Size determination with LDA value > 2.5 along the GI‐tract (p < 0.01). Figure S3: The short‐chain fatty acids (SCFAs) concentrations varied in different intestine segments of Jinhua pigs. Figure S4: The correlation analysis between identified top10 genus bacteria with the short‐chain fatty acids (SCFAs) levels in the different intestine segments, including duodenum, jejunum, ileum, colon, and cecum. Figure S5: The differentially represented metabolic pathways at the Kyoto Encyclopedia of Genes and Genomes (KEGG) level 3 through Linear Discriminant Analysis (LDA) Effect Size determination with LDA value > 2.5 and p < 0.01 along the GI‐tract based on the PICRUSt2. Figure S6: Short‐chain fatty acids (SCFAs) producing related gene expression along the GI‐tract. Figure S7: The functional microbiome profiles of gut microbiome between high and low fatness pigs through functional annotation of metagenome with the Kyoto Encyclopedia of Genes and Genomes (KEGG) database. Figure S8: The Carbohydrate‐Active enZymes (CAZymes) distribution in groups. Figure S9: Network of co‐occurring Carbohydrate‐Active enZymes (CAZymes) based on correlation analysis in the H (n = 7, left panel) and L (n = 7, right panel) fatness pigs. Figure S10: Significant changes of bins between two groups. Figure S11: Phylogenetic distribution of sequences in glycoside hydrolase family 13 (GH13) assigned to the identified bacteria. Figure S12: Carbohydrate‐active enzymes distribution in Clostridium butyricum. Figure S13: qPCR confirmed the successful colonization of Clostridium butyricum in obese mice. Figure S14: Short‐chain fatty acids (SCFAs) levels in the colon of the two groups. [file IMT2-3-e160-s001.docx]

**Supporting information to**

***Clostridium butyricum* and carbohydrate active enzymes contribute to the reduced fat deposition in pigs**

**Running title：*Clostridium butyricum* reduced fat deposition in pigs**

Lingyan Ma^1#^, Shiyu Tao^2#^, Tongxing Song^2#^, Wentao Lyu^1^, Ying Li^3^, Wen Wang^1^, Qicheng Shen^1^, Yan Ni^4^, Jiang Zhu^1^, Jiangchao Zhao^5*^, Hua Yang^1*^, Yingping Xiao^1*^

^1^State Key Laboratory for Managing Biotic and Chemical Threats to the Quality and Safety of Agro-products, Institute of Agro-product Safety and Nutrition, Zhejiang Academy of Agricultural Sciences, Hangzhou, China

^2^College of Animal Sciences and Technology, Huazhong Agricultural University, Wuhan, China 430070

^3^Guangdong Provincial Key Laboratory of Animal Molecular Design and Precise Breeding, College of Life Science and Engineering, Foshan University, Foshan, China

^4^The Children's Hospital, Zhejiang University School of Medicine, National Clinical Research Center for Child Health, Hangzhou 310052, China.

^5^Department of Animal Science, Division of Agriculture, University of Arkansas, Fayetteville, Arkansas, USA

^#^ These authors contributed equally: Lingyan Ma, Shiyu Tao, Tongxing Song

^*^Corresponding author:

xiaoyp@zaas.ac.cn (Yingping Xiao)

yanghua@zaas.ac.cn (Hua Yang)

[jzhao77@uark.edu](mailto:jzhao77@uark.edu) (Jiangchao Zhao)

**Supplementary Method**

**Short-Chain Fatty Acids (SCFAs) analysis**

Each intestinal content sample was mixed with 10 mL/g of deionized water, vortex for 1 min, mix thoroughly until no obvious clumping. The mixtures were then centrifuged (10,000 rpm for 10 min). Use a pipette to transfer 1 ml of supernatant into a centrifuge tube. Add 200 μL of crotonic acid (internal standard for GC, ≥ 99.8%, Merck, USA) to the supernatant, invert the centrifuge tube to completely mix the supernatant. Then, centrifuge the sample at 10,000 rpm for 10 min. Aspirate the supernatant with disposable sterile syringe and filter through a 0.22 μm organic filter membrane. Finally, the mixed solution was used to determine the concentration of SCFAs using Gas Chromatography (GC) systems (Shimadzu, Japan) after filtering through a membrane filter as previously described [1]. The GC program were as followed: inlet temperature: 250 °C; Injection volume: 1 µl; Shunting ratio: 1:1; Carrier gas type: N2; Carrier gas velocity: 2 mL/min; FID detector: 250 °C; Flow of the H2: 40 ml/min; Flow of the synthetic air: 450 ml/min.

**Bacterial 16S rRNA gene sequencing**

Bacterial DNA from the different intestinal tracts of the 14 samples (H group and L group) from animal trial 1, and 36 samples (Control group and CB group) from animal trial 3, respectively, were isolated from each intestinal digesta using the QIAamp DNA Stool Mini Kit (QIAGEN, CA, USA) according to the manufacturer's instructions. The V4-V5 hypervariable region of the bacterial 16S rRNA gene was then amplified with degenerate primers, 515 F (5-GTGCCAGCMGCCGCGG-3) and 907 R (5-CCGTCAATTCMTTTRAGTTT-3) [2]. For library preparation, 1 μg of genomic DNA from each sample was used for PCR amplification. Amplicons from these samples were purified and normalized before being pooled in equal amounts for library preparation that was sequenced with Illumina’s TruSeq platform. Libraries were generated using the Illumina TruSeq DNA PCR-Free Library preparation kit and sequenced by Novogene (Beijing, China) on an Illumina HiSeq 4000. Raw reads were first filtered for quality before being merged into tags using FLASH, which were then assigned to each sample according to the unique barcodes. Raw data were analyzed using the QIIME2 platform. PCA ordination analysis was conducted by Bray-Curtis distance matrices based on the relative abundance table of the taxonomic composition (R Package ade4, v1.7.13). Phylogenetic Investigation of Communities by Reconstruction of Unobserved States (PICRUSt2) was used to predict the functional capabilities of the bacterial community as described [3].

**Functional annotation**

Functional annotation of the "non-redundant" gene catalog was performed using BLASTP against the KEGG database (https://www.genome.jp/kegg/) to obtain KEGG Orthologs (KO) with an e-value of 10^-5^ [4]. Then, we used custom Perl scripts to gain the abundance PPM (Part Per Million, one KO pathway-assigned sequence per million sequences) of KO pathways for each metagenome. LEfSe was used to identify the significant difference in the abundance of KEGG pathways between high and low fatness pigs based on the LDA > 3, *p* value < 0.05. CAZymes were annotated by using HMMER (v.3.2.1) to match protein sequences to entries in the hidden Markov model (HMM) libraries of CAZyme families downloaded from the CAZyme database (v.7; http://www.cazy.org/) [5,6] (Table S4). Circos was used to visualize the contribution of bacteria taxon regarding the CAZyme families based on the PPM (part per million, one CAZyme-like sequence per million sequences) of bacterial genera for the annotated CAZyme families. The correlation analysis was performed by Spearman's correlation analysis. Gephi (v.0.9.1) was utilized to visualize the network of correlations between CAZyme families. The significance of CAZyme families between the high and low fatness groups was determined using Welch's t-test and Benjamini-Hochberg FDR correction available in STAMP software [7]. The heatmap was generated using TB tools software (a Toolkit for Biologists integrating various biological data-handling tools).

**Isolation and culture of the bacterial strain of *C. butyricum***

The fecal samples from experimental Jinhua pigs with low fatness phenotypic values and a high abundance of *C. butyricum* were collected and used for the *C. butyricum* mouse intervention experiment. One gram of each fecal sample was suspended in phosphate-buffered saline (PBS) buffer and serially diluted to 10^−8^. Reinforced Clostridial Medium (RCM medium) (Thermo Fisher Scientific Inc., USA) was used, which consisted of 5 g glucose, 3 g yeast extract, 10 g beef extract, 10 g tryptone, 1 g soluble starch, 5 g NaCl, 3 g sodium acetate trihydrate, 0.5 g cysteine hydrochloride, and 12.5 g agar (used with solid medium) and 1,000 mL distilled water at pH 6.5-7.0. The procedure for bacteria isolation was as follows: (1) 1 mL aliquot of the enriched culture was aseptically moved to RCM solid medium and incubated under anaerobic conditions for 48 h at 37 °C; (2) single colonies on the solid medium were selected, and separately mixed into deoxidized sterile normal saline; (3) 0.5 mL of the bacterial suspension was inoculated on fresh solid medium and incubated anaerobically for 48 h at 37 °C; (4) operation (2) and (3) were repeated twice to obtain the pure cultures of isolates. The 16S rDNA gene sequence of each isolate was compared with other reference sequences available in the NCBI database using the Basic Local Alignment Search Tool (BLAST) algorithm.

**RNA extraction and RT-PCR**

The total RNA from the adipose tissues was extracted by using TRIzol (Vazyme Biotech Co., Ltd). RT-PCR was performed with 2×ChamQ SYBR Color qPCR Master Mix (Vazyme Biotech Co., Ltd.) according to the manufacturer’s instructions. Relative mRNA expressions were quantified using the threshold cycle (2^−ΔΔCT^) method as described previously [9]. The primers are shown in Table S9.

**Quantitation for *C. butyricum* in treated mice**

Mouse colonic bacterial DNA was extracted using the QIAamp fast DNA stool mini kit (Qiagen, Hilden, Germany) according to the manufacturer's instructions. The two-step real-time PCR conditions were described as follows: an initial denaturation for 15 s at 95 °C, 40 cycles of denaturation at 95 °C for 5-10 s, and annealing at 60 °C for 5-15 s, and elongation at 72℃ for 20 s. The relative quantification (RQ) value of *C. butyricum* was determined by normalization to the 16S rRNA gene using the 2^−ΔΔCt^ method. Primer sequences are listed in Table S10.

**Supplementary Figures**

**
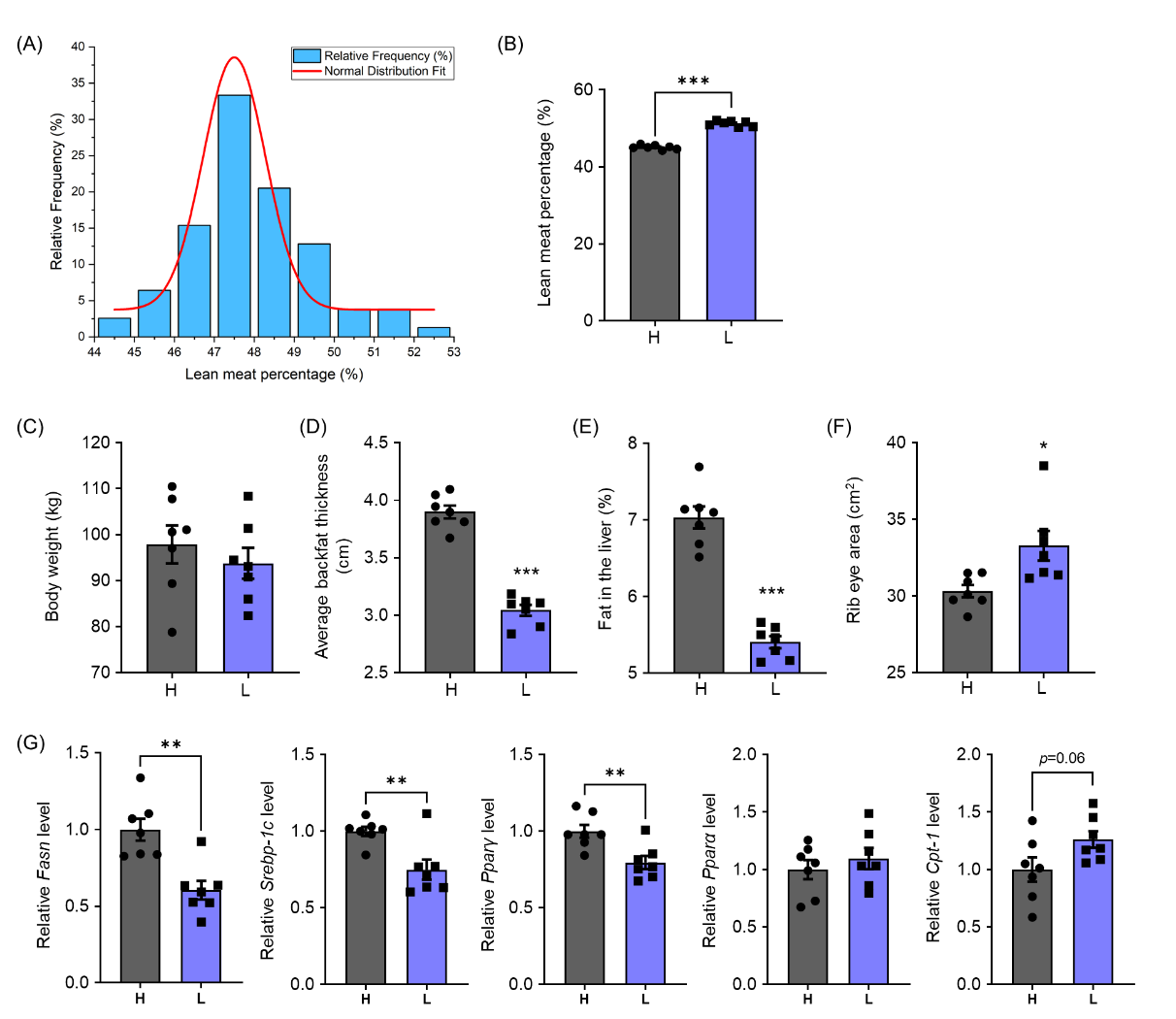
**

**Figure S1.** **The fatness phenotypes of pigs (*n* = 7).** (A) The distribution of phenotypic values of lean meat percentage for the first animal trail. The phenotypic values of lean meat percentage obey a normal distribution. (B) Lean meat percentage in high fatness Jinhua pigs (H) and low fatness Jinhua pigs (L) groups. (C) Body weight. (D) Average backfat thickness. (E) Fat in the liver. (F) Rib eye area. (G) The expression of lipogenesis and lipolysis related genes in subcutaneous adipose tissue. Data were presented as the means ± SEM, **p* < 0.05, ***p* < 0.01, ****p* < 0.001. *Srebp-1c*, Sterol regulatory element binding protein-1; *Pparg*, Peroxisome Proliferator Activated Receptor Gamma; *Fasn*, Fatty acid synthase; *Ppara*, Peroxisome Proliferator Activated Receptor Alpha; *Cpt-1a*, Carnitine palmitoyltransferase 1A.

**
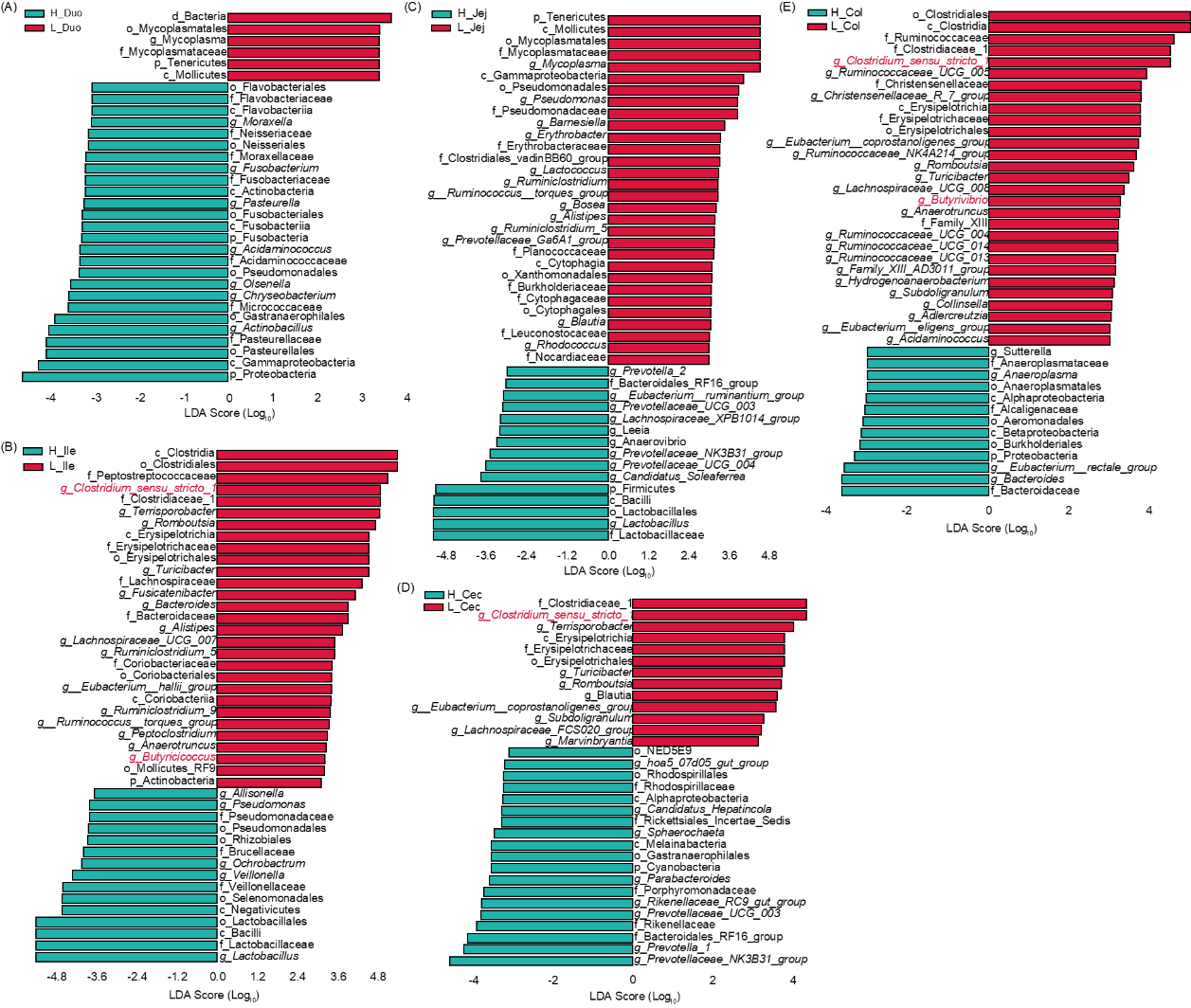
**

**Figure S2.** **The differentially represented bacterial communities through Linear Discriminant Analysis (LDA) Effect Size determination with LDA value > 2.5 along the GI-tract (*p* < 0.01).** (A) Duodenum. (B) Ileum. (C) Jeunum. (D) Colon. (E) Cecum.

**
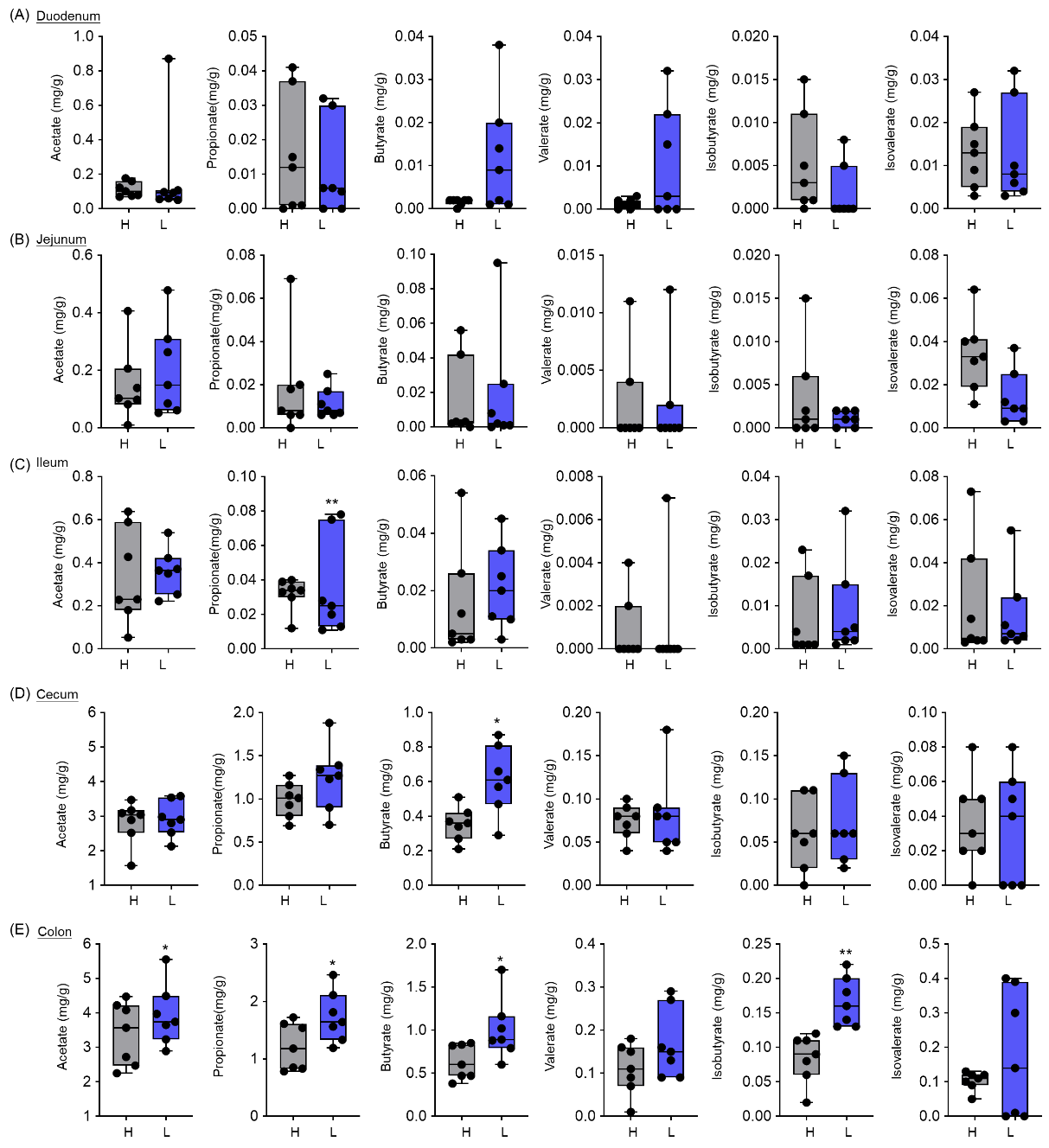
**

**Figure S3.** **The short-chain fatty acids (SCFAs) concentrations varied in different intestine segments of Jinhua pigs.** The production of SCFAs in the (A) Duodenum. (B) Jejunum. (C) Ileum. (D) Cecum and (E) Colon.

**
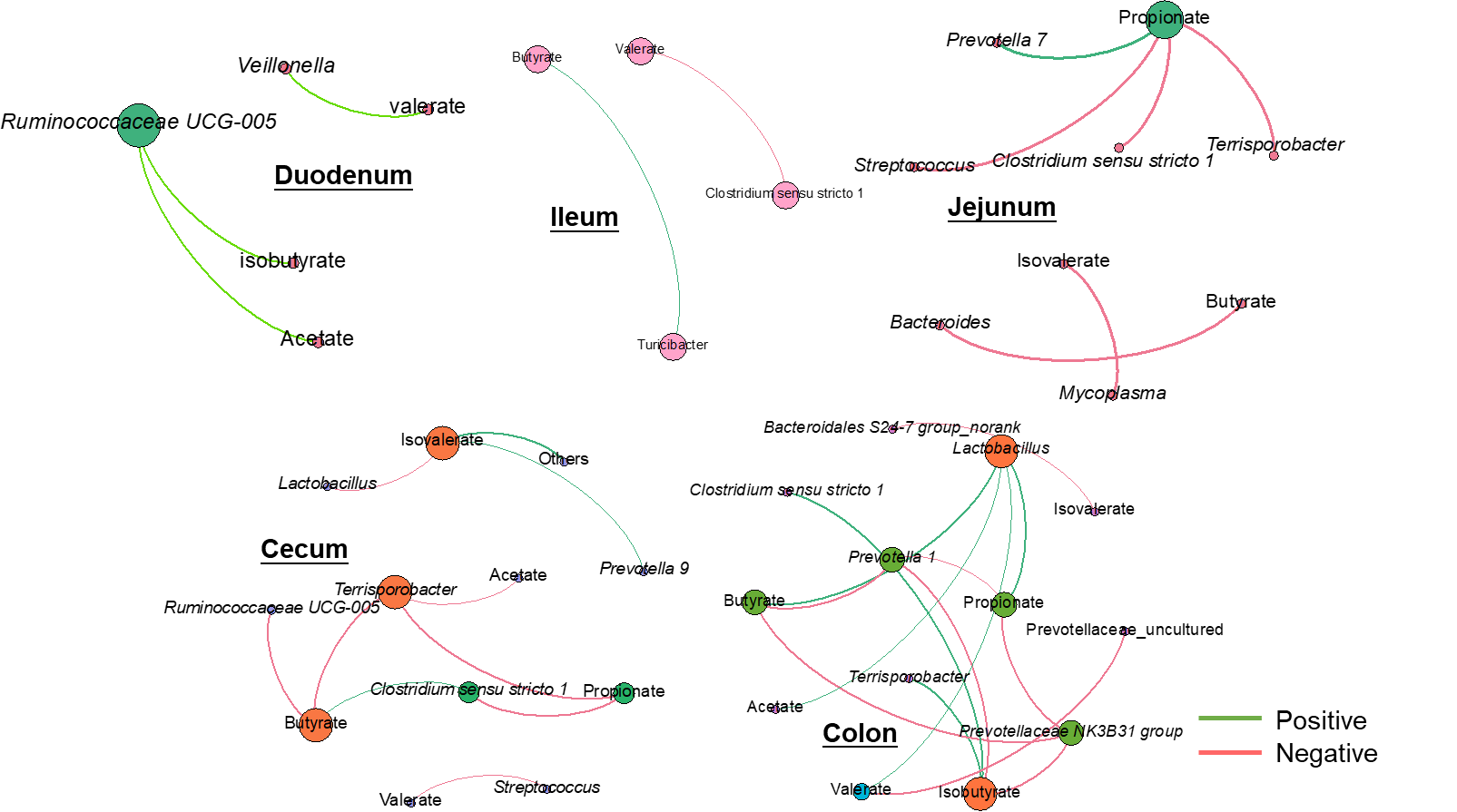
**

**Figure S4.** **The correlation analysis between identified top10 genus bacteria with the short-chain fatty acids (SCFAs) levels in the different intestine segments, including duodenum, jejunum, ileum, colon, and cecum.** The Spearman correlation coefficient reveals the association between the changes in bacteria genera and the SCFAs production in the different intestine segments (corr > 0.5; *p*< 0.05). The lines’ colors represent two kinds of correlation: green for positive correlation and red for negative correlation.

**
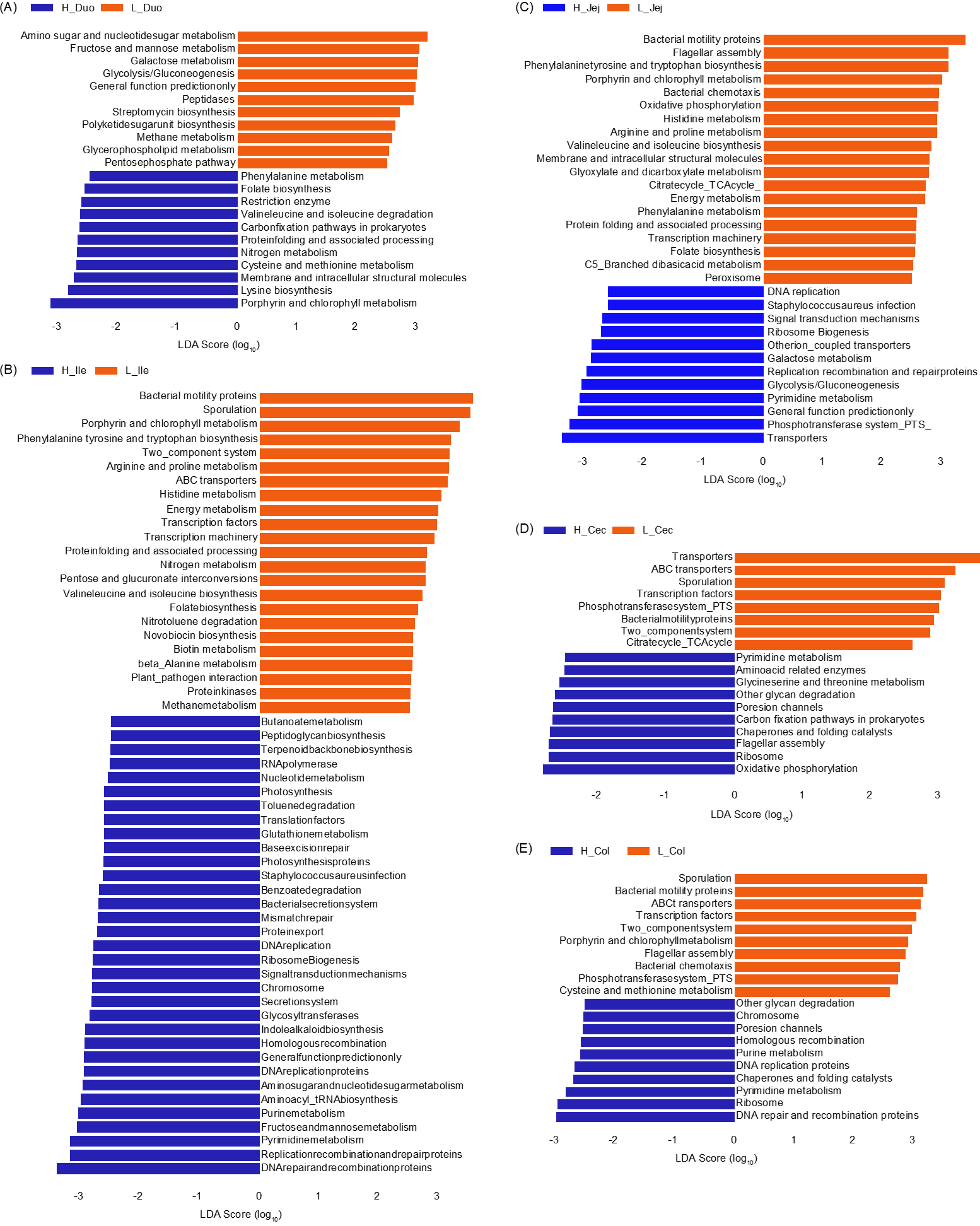
**

**Figure S5.** **The differentially represented metabolic pathways at the Kyoto Encyclopedia of Genes and Genomes (KEGG) level 3 through Linear Discriminant Analysis (LDA) Effect Size determination with LDA value > 2.5 and *p* < 0.01 along the GI-tract based on the PICRUSt2.** (A) Duodenum. (B) Ileum. (C) Jejunum. (D) Colon. (E) Cecum.


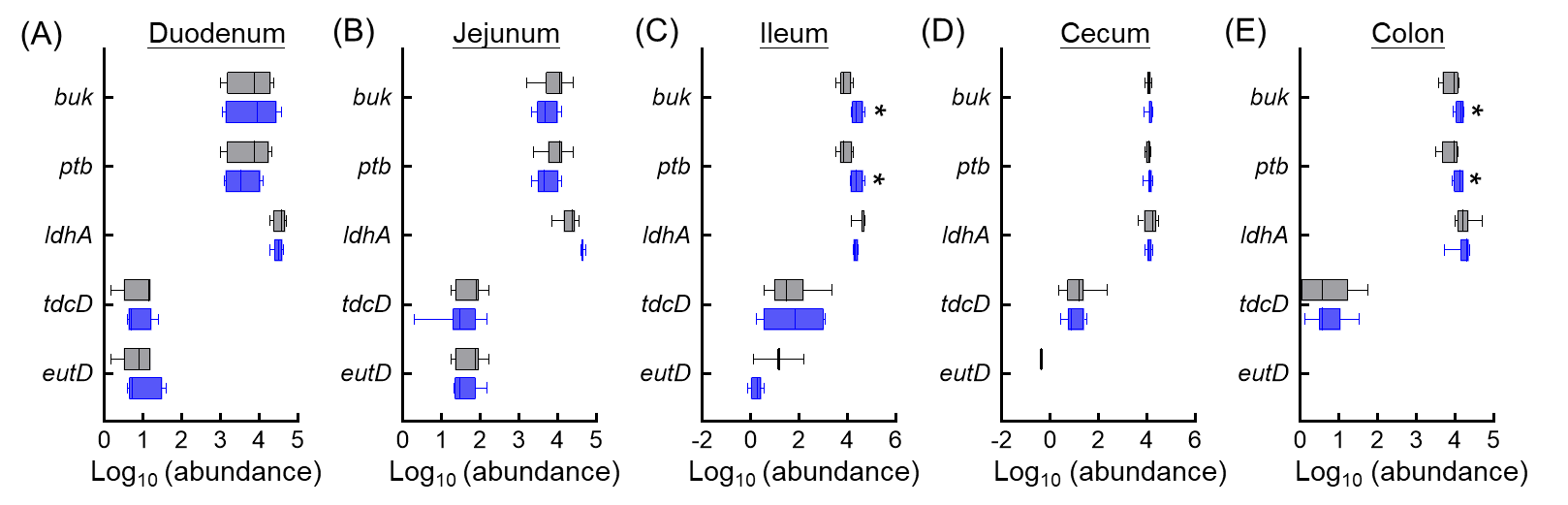


**Figure S6.** **Short-chain fatty acids (SCFAs) producing related gene expression along the gastrointestinal tract (GI)-tract**. (A) Duodenum. (B) Ileum. (C) Jejunum. (D) Colon. (E) Cecum. Buk, butyrate kinase; ptb, phosphate butyryltransferase; ldhA, D-lactate dehydrogenase; tdcD, propionate kinase; eutD phosphotransacetylase.


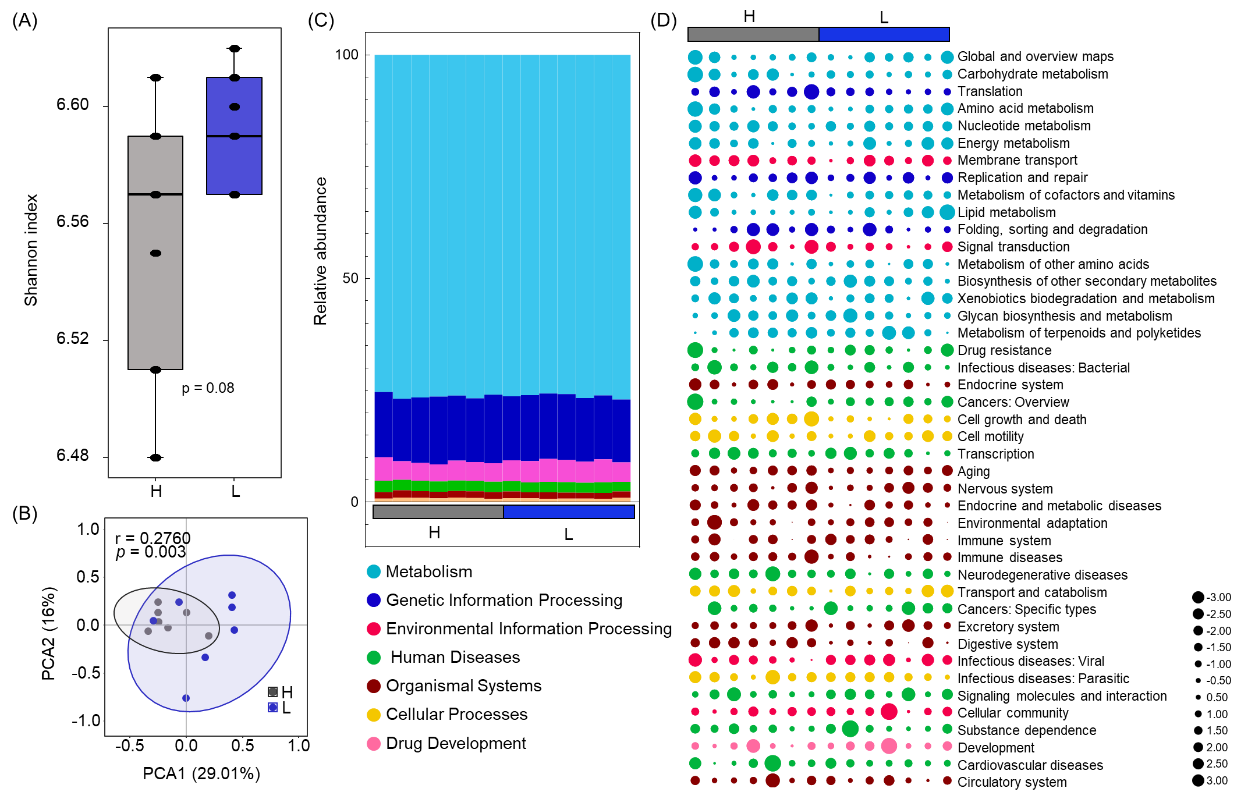


**Figure S7. The functional microbiome profiles of gut microbiome between high and low fatness pigs through functional annotation of metagenome with the Kyoto Encyclopedia of Genes and Genomes (KEGG) database.** (A) Shannon index between two groups. (B) Principal coordinate analysis (PCoA) plots based on Bray-Curtis and Jaccard distances. (C) KEGG function at Class levels. (D) KEGG function at Subclass levels.

**
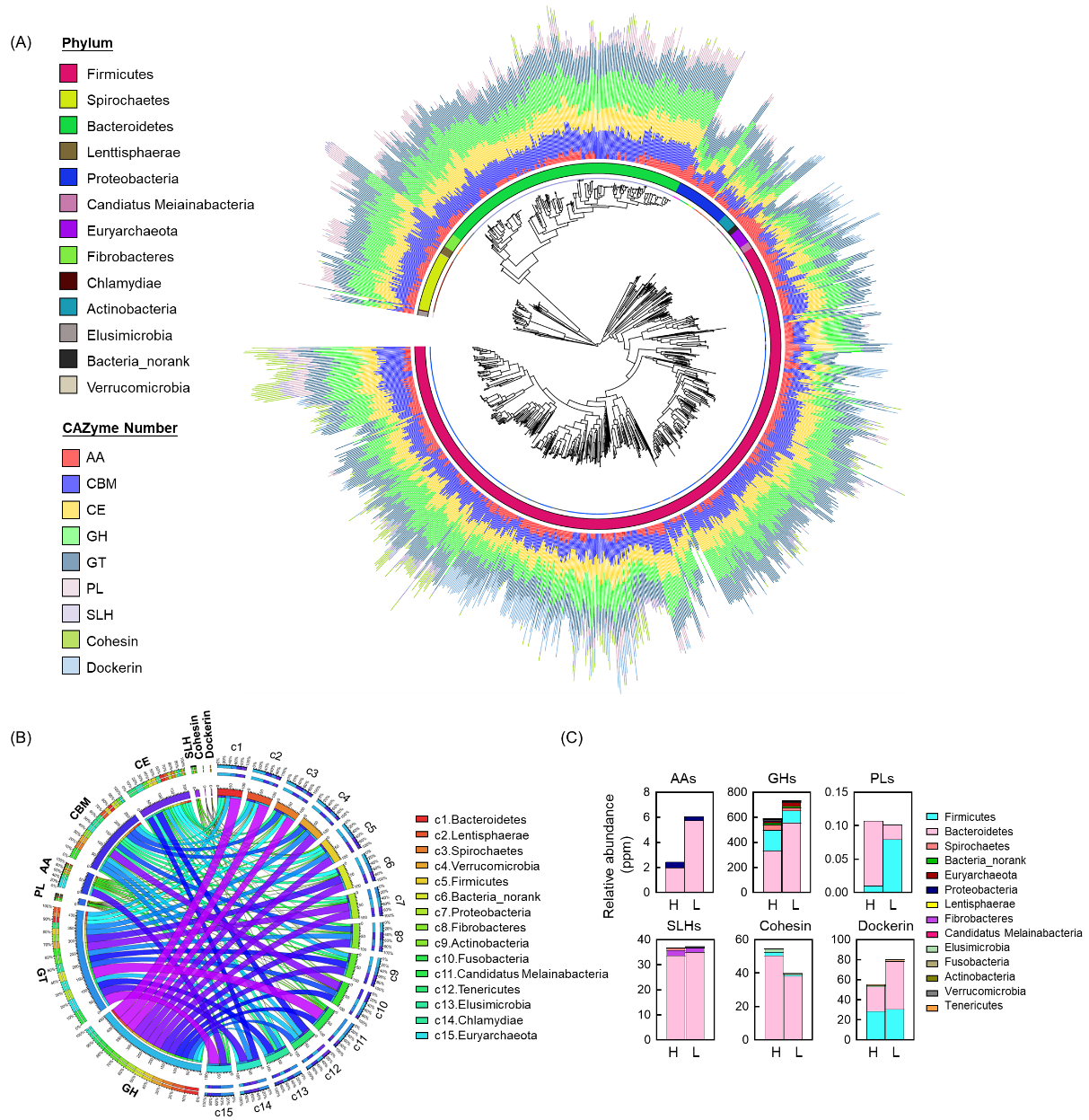
**

**Figure S8. The Carbohydrate-Active enZymes (CAZymes) distribution in groups.** (A) Phylogenetic tree of the reconstructing 1288 metagenome-assembled genomes (MAGs) shows the encoded CAZymes number in each MAGs. The outermost stacked bar chart depicts the abundance of different CAZyme classes encoded in each MAG. The different colors of the outer circle represent the phylum-level classification of these MAGs. (B) The proportions of CAZymes assigned to each CAZymes module within MAGs classified in phylum level. (C) The above distribution of each CAZymes class assigned to the identified phyla in the high fatness (H) and low fatness (L) groups. GH, Glycoside hydrolase family. AA: Auxiliary Activities; CBM: Carbohydrate-binding modules; GH: Glycoside hydrolases; GT: Glycosyl Transferase; PL: Polysaccharide lyase; SLH: S-layer homology domain; PTS: Phosphotransferase system; CE: Carbohydrate Esterase.

**
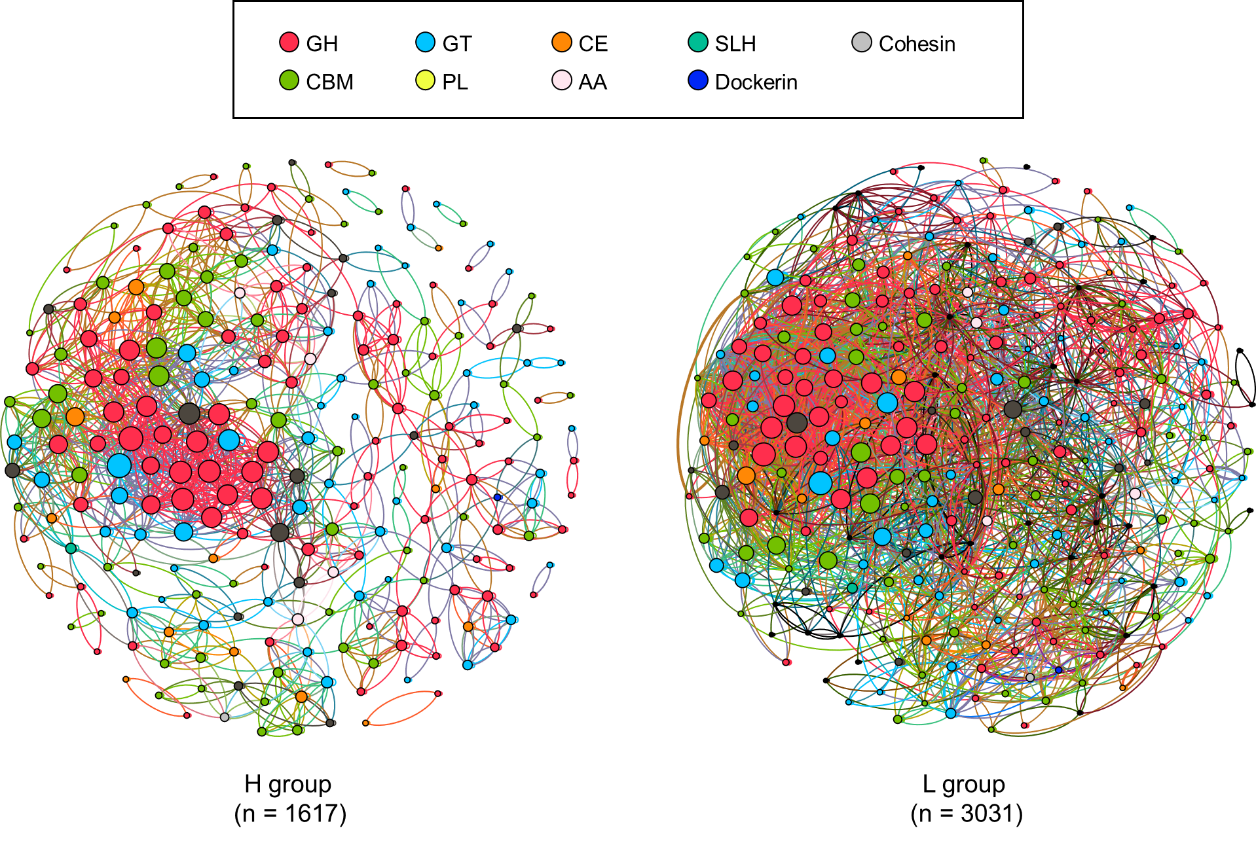
**

**Figure S9. Network of co-occurring Carbohydrate-Active enZymes (CAZymes) based on correlation analysis in the high fatness (H, *n* = 7, left panel) and low fatness (L*, n* = 7, right panel) pigs.** A connection stands for a strong (Spearman's ρ>0.6) and significant (*p*-value <0.01) correlation. The size of each node is proportional to the number of connections. CAZymes were colored by different classes.


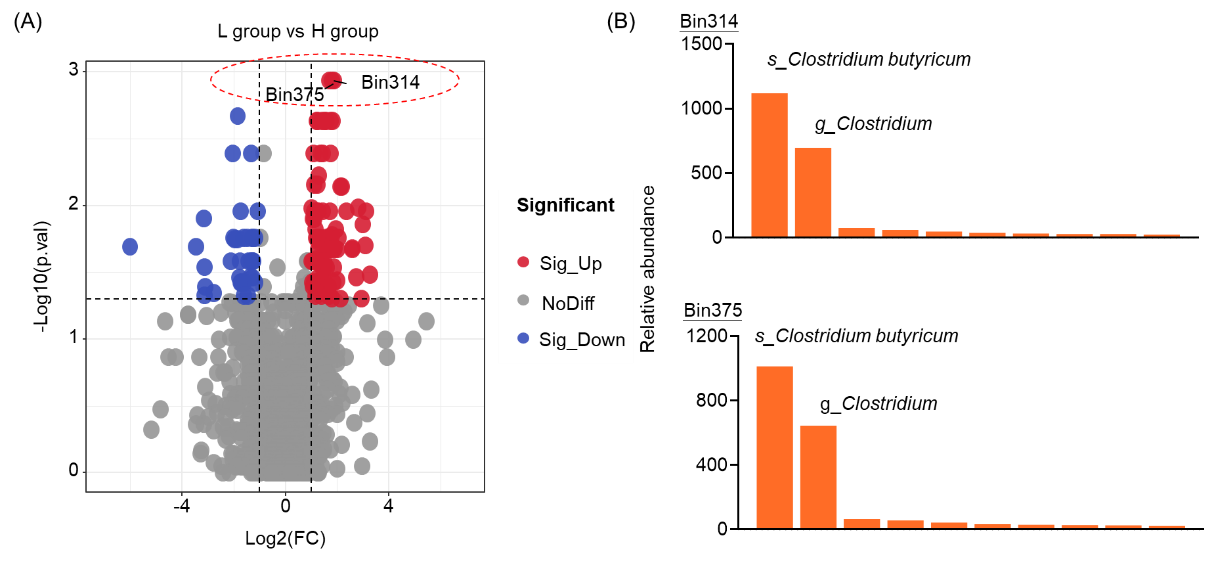


**Figure S10. Significant changes of bins between two groups.** (A) Volcano plot showing the identified most biologically significant bins with large fold change that are also statistically significant, and The high-quality Bin314 and Bin375 were significantly up-regulated in the L group. (B) The identified Bin314 and Bin375.


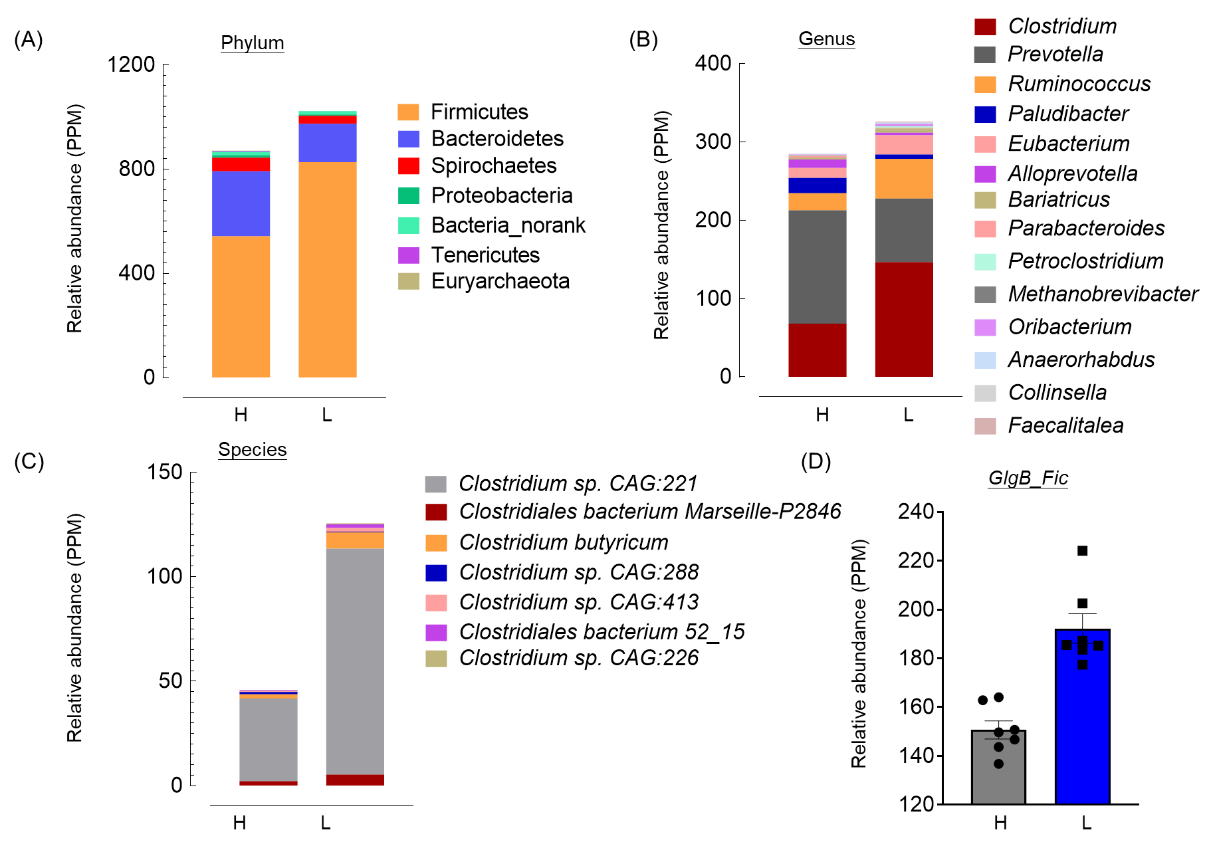


**Figure S11. Phylogenetic distribution of sequences in glycoside hydrolase family 13 (GH13) assigned to the identified bacteria.** (A) Phylum level. (B) Genus levels, (C) Species levels. (D) The α-(1 → 4)-glucanotransferase GlgB abundance in the two groups.


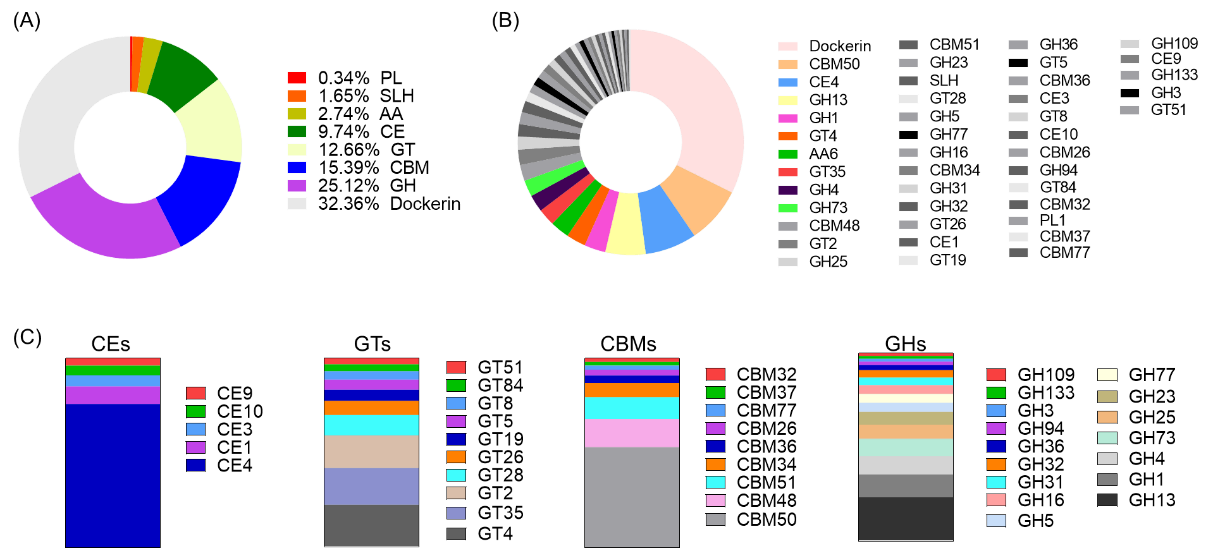


**Figure S12.** **Carbohydrate-active enzymes distribution in *Clostridium butyricum*.** (A) Distribution of major CAZyme class in *C. butyricum* genomes, (B) Distribution of major CAZyme families in *C. butyricum* genomes, (C) Families belong to CE, GT, CBM and GH in *C. butyricum* genomes. CBM: Carbohydrate-binding modules; GH: Glycoside hydrolases; GT: Glycosyl Transferase; CE: Carbohydrate Esterase.

**
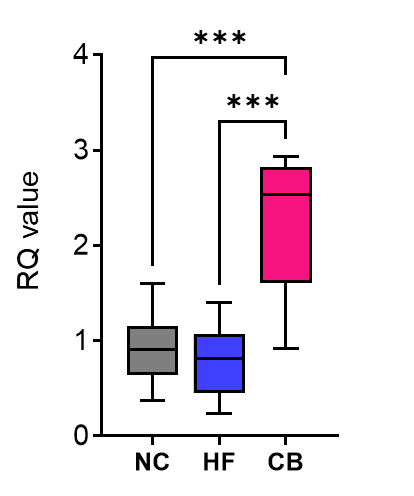
**

**Figure S13. qPCR confirmed the successful colonization of *Clostridium butyricum* in obese mice.** The Y-axis indicates the Relative quantification (RQ) values reflecting the relative abundance of *C. butyricum* in treated mice.


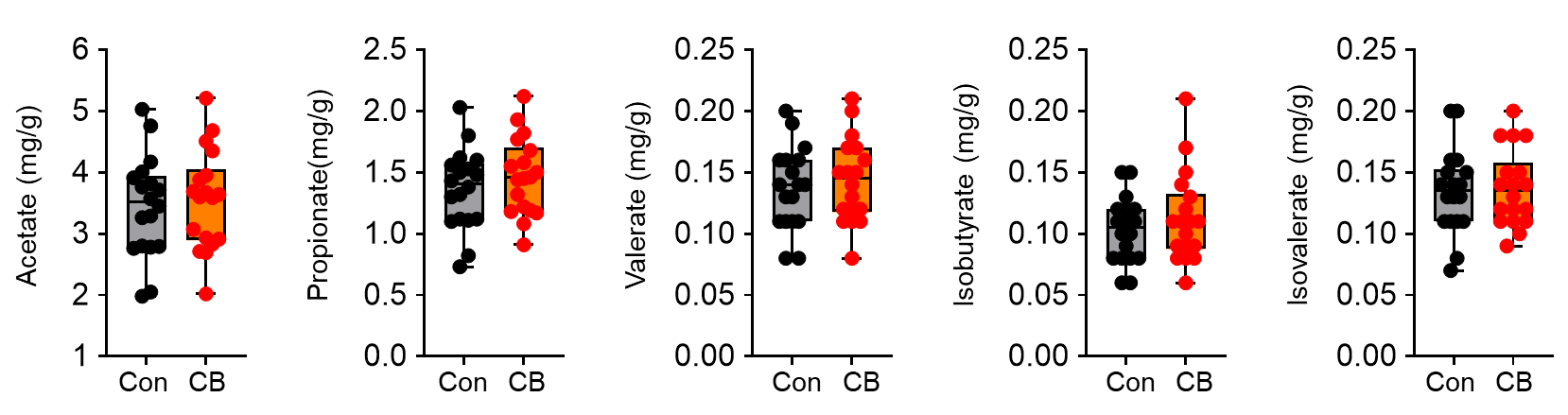


**Figure S14. Short chain fatty acid levels in the colon of the two groups.** Data were presented as the means ± SEM.

**REFERENCES**

1. Yang, Hua, Yingping Xiao, Junjun Wang, Yun Xiang, Yujie Gong, Xueting Wen, Defa Li. 2018. “Core gut microbiota in Jinhua pigs and its correlation with strain, farm and weaning age.” *Journal of Microbiology* 56: 346−355. <https://doi.org/10.1007/s12275-018-7486-8>

2. Xiao, Yingping, Fanli Kong, Yun Xiang, Weidong Zhou, Junjun Wang, Hua Yang, Guolong Zhang, et al. 2018. “Comparative biogeography of the gut microbiome between Jinhua and Landrace pigs.” *Scientific Repeports* 8: 5985. <https://doi.org/10.1038/s41598-018-24289-z>

3. Yang, Chen, Jiahao Mai, Xuan Cao, Aaron Burberry, Fabio Cominelli, Liangliang Zhang. 2023. “ggpicrust2: an R package for PICRUSt2 predicted functional profile analysis and visualization.” *Bioinformatics* 39: btad470. <https://doi.org/10.1093/bioinformatics/btad470>

4. De Filippo, Carlotta, Matteo Ramazzotti, Paolo Fontana, Duccio Cavalieri. 2012. “Bioinformatic approaches for functional annotation and pathway inference in metagenomics data.” *Briefing in Bioinformatics* 13: 696−710. <https://doi.org/10.1093/bib/bbs070>

5. Potter, Simon C, Aurélien Luciani, Sean R Eddy, Youngmi Park, Rodrigo Lopez, Robert D Finn. 2018. “HMMER web server: 2018 update.” *Nucleic Acids Research* 46: W200−W204. <https://doi.org/10.1093/nar/gky448>

6. Lombard, Vincent, Hemalatha Golaconda Ramulu, Elodie Drula, Pedro M Coutinho, Bernard Henrissat. 2014. “The carbohydrate-active enzymes database (CAZy) in 2013.” *Nucleic Acids Res* 42: D490−495. <https://doi.org/10.1093/nar/gkt1178>

7. Parks, Donovan H, , Gene W Tyson, Philip Hugenholtz, Robert G Beiko. 2014. “STAMP: statistical analysis of taxonomic and functional profiles.” *Bioinformatics* 30: 3123−3124. <https://doi.org/10.1093/bioinformatics/btu494>

8. Chen, Chengjie, Hao Chen, Yi Zhang, Hannah R Thomas, Margaret H Frank, Yehua He, Rui Xia. 2020. “TBtools: An Integrative Toolkit Developed for Interactive Analyses of Big Biological Data.” *Molecular Plant* 13: 1194−1202. <https://doi.org/10.1016/j.molp.2020.06.009>

9. Ma, Lingyan, Qicheng Shen, Wentao Lyu, Lu Lv, Wen Wang, Minjie Yu, Hua Yang, et al. 2022. “*Clostridium butyricum* and Its Derived Extracellular Vesicles Modulate Gut Homeostasis and Ameliorate Acute Experimental Colitis.” *Microbiology Spectrum* 10: e0136822. <https://doi.org/10.1128/spectrum.01368-22>
